# Anabaenopeptins: What We Know So Far

**DOI:** 10.3390/toxins13080522

**Published:** 2021-07-27

**Authors:** Patrick Romano Monteiro, Samuel Cavalcante do Amaral, Andrei Santos Siqueira, Luciana Pereira Xavier, Agenor Valadares Santos

**Affiliations:** 1 Laboratory of Biotechnology of Enzymes and Biotransformation, Biological Sciences Institute, Federal University of Pará, Belém 66075-110, Brazil; samuel.amaral@icb.ufpa.br (S.C.d.A.); lpxavier@ufpa.br (L.P.X.); 2 Laboratory of Biomolecular Technology, Biological Sciences Institute, Federal University of Pará, Belém 66075-110, Brazil; andrei.siqueira@icb.ufpa.br

**Keywords:** cyanobacteria, peptide, NRPS, anabaenopeptin

## Abstract

Cyanobacteria are microorganisms with photosynthetic mechanisms capable of colonizing several distinct environments worldwide. They can produce a vast spectrum of bioactive compounds with different properties, resulting in an improved adaptative capacity. Their richness in secondary metabolites is related to their unique and diverse metabolic apparatus, such as Non-Ribosomal Peptide Synthetases (NRPSs). One important class of peptides produced by the non-ribosomal pathway is anabaenopeptins. These cyclic hexapeptides demonstrated inhibitory activity towards phosphatases and proteases, which could be related to their toxicity and adaptiveness against zooplankters and crustaceans. Thus, this review aims to identify key features related to anabaenopeptins, including the diversity of their structure, occurrence, the biosynthetic steps for their production, ecological roles, and biotechnological applications.

## 1. Introduction

Cyanobacteria are photosynthetic microorganisms widely distributed in the world. They can inhabit several types of ecosystems, including aquatic and terrestrial. These microorganisms produce a great variety of bioactive compounds, which have been investigated mainly due to their biotechnological potential and environmental relevance [1,2,3]. Cyanotoxins are among the most studied compounds originated from cyanobacteria since they are capable of negatively affecting human and animal health [4,5]. These metabolites can vary drastically concerning their action mechanism and chemical structure, which include peptides, alkaloids, and lipopolysaccharides [6,7,8]. The majority of publications related to peptides from cyanobacteria have mainly focused on the class of microcystins with over 300 characterized variants [9,10]. However, cyanobacteria usually do not exclusively produce a single class of compounds, given that specific strains are co-producing different groups of secondary metabolites [11].

Other peptides beyond microcystins have been poorly explored, lacking information mainly in the environmental sciences [11]. These several metabolites are known for their potent inhibitory properties against several enzymes in nanomolar concentrations, resulting in toxic effects [12,13]. Moreover, similar to microcystins, they have been regularly detected in diverse environments [14]. In certain regions, their occurrence is more pronounceable than microcystins themselves [15]. However, information about the concentrations which are encountered is rarely reported [11]. Cyanobacteria have developed different peptides as a protection mechanism against parasites [16]. Concerning their origin, some peptides as microviridins and cyanobactins are produced via ribosomal whereas others as microginins and aeruginosins are synthesized by non-ribosomal pathways [13,17,18].

Among the most recurrent peptides encountered in the environment are anabaenopeptins (APs), a family of cyclic peptides containing six amino acid residues [19]. They have been found in an enormous variety of cyanobacteria isolated from both the aquatic and terrestrial environments, including *Anabaena*, *Nostoc*, *Microcystis*, *Planktothrix*, *Lyngbya,* and *Brasilonema* [12,20,21,22,23,24]. In their general structure is a well-conserved Lysine (Lys) residue in D-configuration, which is responsible for the ring formation and five additional variable amino acids, either proteinogenic or non-proteinogenic, resulting in 124 described AP variants from cyanobacteria (Supplementary Table S1). [19]. Besides their structural variety, molecules belonged to this group exhibit an impressive functional diversity, which includes inhibitory activity for proteases, phosphatases, and carboxypeptidases [22,25,26].

The enormous structural diversification of anabaenopeptins can be attributed to the low substrate specificity of some enzymes involved in their synthesis as well as the presence of alternative starter modules [16]. Their production is strongly influenced by environmental factors [27]. Besides that, because of their diversified bioactive properties, they exhibit an elevated biotechnological potential. This review aims at presenting the main researches on anabaenopeptins, emphasizing their general characteristics, biosynthesis as well as ecological and biotechnological relevance.

## 2. Structures of Anabaenopeptins

**Figure 1:**
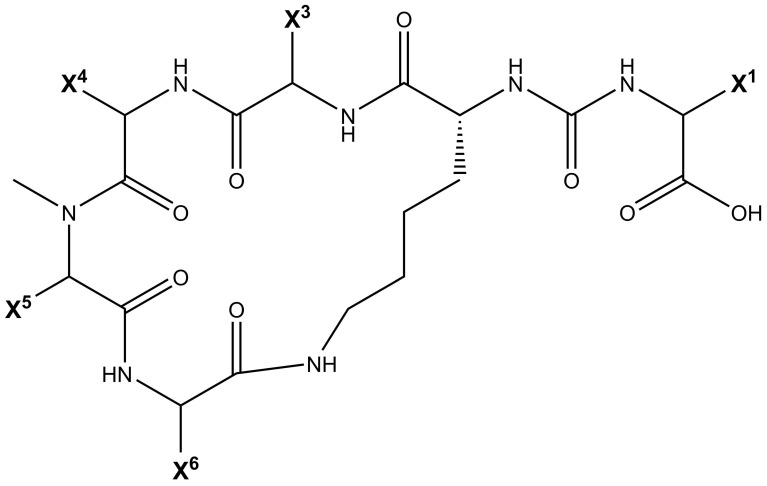
The general structure of the class of Anabaenopeptins. X corresponds to different amino acids in their respective positions represented by the superscript numbers.

Being non-ribosomally synthesized, anabaenopeptin structures comprise a ring of five amino acids connected through an ureido linkage to an exocyclic amino acid. Thus, its general structure is represented by X^1^-CO-[Lys^2^-X^3^-X^4^-MeX^5^-X^6^], where the bracket represents the cyclic region of this peptide and X are variable amino acids according to their positions represented by the superscript numbers (Figure 1). Its ring is formed by cyclization of the C-terminal carboxyl of the amino acid at position 6 to the ε-NH_3_ of the well-conserved D-lysine at position 2. Furthermore, the α-amino group of Lys is connected to the exocyclic amino acid X^1^ via an ureido bridge. Due to its non-ribosomal nature, proteinogenic and non-proteinogenic amino acids are usually detected in this hexapeptide [19].

This family of peptides is predominantly found in cyanobacteria, but they were also detected in some sponges [28,29,30,31,32,33]. However, Anabaenopeptins from cyanobacterial origins demonstrate a well-conserved D-Lys, while the other amino acids are in L configuration and vary in residues and modifications (e.g., acetylation, methylation) [19,34]. In comparison, Anabaenopeptins derived from sponges can have both D- and L-configuration of Lys residues at the second position. Besides these differences, some features in common are also encountered, such as the frequently N-methylated amino acids at position 5 and homo-amino acids at position 4. However, exceptions are also found, for example, Paltolides A–C, a subgroup of anabaenopeptin-like peptides that have in common a tryptophan residue at the C-terminus linked to ε-amine of the N-terminal Lys, and Leucine (Leu) in L-configuration at positions 4 and 5, and an L-Alanine (Ala) residue at position 3. Furthermore, Paltolide A is the first example of this class of peptides where the amino acid at position 5 lacks an N-methyl group [28,29,30,31,32,33].

**Figure 2:**
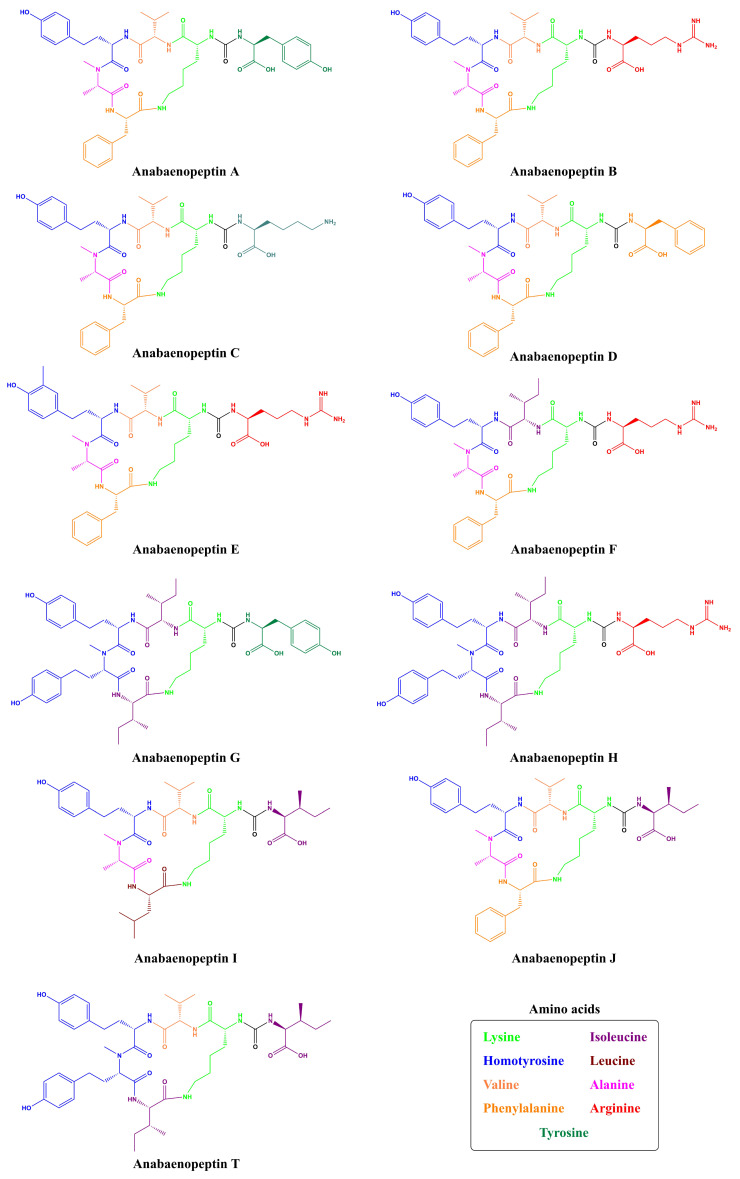
Structures of anabaenopeptins A–J [20,26,35,38,42] and T [41].

The first Anabaenopeptins detected were Anabaenopeptins A and B (Figure 2; Supplementary Table S1) by Harada and co-workers in 1995 [20]. These peptides were isolated from *Anabaena flos-aquae* NRC 525-17, where they were co-produced with Microcystins (MCs) and the neurotoxic alkaloid anatoxin-A. Both peptides share the same cyclic sequence and structure, differing only at the exocyclic position: (Arginine/Tyrosine)-Lys-Valine-Homotyrosine-NMethylAla-Phenylalanine. Due to their origin, these peptides were then named after their producer. Following the first detection of this new peptide, Fujii and co-workers [35] identified Anabaenopeptins A-D from different *Anabaena* and *Oscillatoria* strains, as well as from *Nodularia spumigena*. APs C and D differ solely by the exocyclic amino acid, harboring a Lys and a Phenylalanine (Phe), respectively, and sharing the same pentapeptide ring with APs A and B. Furthermore, in the same year, Sano and Kaya [36] identified a peptide named Oscillamide Y, which was obtained from *Oscillatoria agardhii* NIES-610. Following the same nomenclature, Sano and colleagues [25] further characterized both Oscillamide B and C (known as Anabaenopeptin F) from *Planktothrix agardhii* CCAP 1459/11A and *P. rubescens* CCAP 1459/14. Besides their different nomenclature, Oscillamides peptides also possess the same common features of anabaenopeptin-peptides.

The cyanobacteria *Oscillatoria agardhii* NIES-204 had been assessed regarding AP production by two different research studies. During the first approach, only Anabaenopeptin B had been detected [37]. Later, Shin and co-workers [38] were able to characterize two new structures from the same organism: Anabaenopeptins E and F, which differ at those residues in positions 3 and 4. Later, two new AP structures were identified in *O*. *agardhii* NIES-595, then named Anabaenopeptins G and H, diverging by Tyrosine (Tyr) and Arginine (Arg) in position 1, respectively: (Tyr/Arg)-Lys-Isoleucine-Homotyrosine-NMethylhomotyrosine-Isoleucine [26].

The first unicellular cyanobacterium strain to be identified as an Anabaenopeptin producer was *Microcystis aeruginosa* Kutz. This freshwater strain was able to biosynthesize the anabaenopeptin-type Ferintoic Acids A and B [39]. Another *M*. *aeruginosa* strain and an environmental sample containing mostly *Microcystis* cells demonstrated to contain the Non-Ribosomal Peptide Synthetase (NRPS) apparatus for Anabaenopeptins B and F production. Also, the same work concluded that the filamentous cyanobacteria *Planktothrix agardhii* HUB011 produced the Anabaenopeptin G [40].

Kodani and co-workers [41] evaluated the presence of anabaenopeptins in an environmental sample from Lake Teganuma. Besides the identification of microginins and micropeptins, a newly found AP was characterized: Anabaenopeptin T. However, this nomenclature did not follow any specific order, as Anabaenopeptin I and J had been only used for the new peptides obtained from *Aphanizomenon flos-aquae* NIES-81 and identified by Muramaki and co-workers [42], one year later from this previous work.

Some of those conserved features from APs can also be visualized in other cyanopeptides. Veraguamides A-G are cyclic hexadepsipeptides, and they do not possess any exocyclic residue. Lyngbyastatin peptides demonstrate elastase, trypsin, and chymotrypsin inhibitory properties. Their structures consist of a 6-member ring coupled to a chain of 2 exocyclic residues and can bear modified and unusual residues. Also possessing a 6-member ring structure and 2 exocyclic amino acids, Tiglicamides A-C were obtained from *Lyngbya confervoides* [43]. 

In addition, there are several classes of toxic peptides frequently detected in cyanobacteria, each one presents the main structure codified by a set of NRPS genes. Besides Anabaenopeptins, Microcystins, Cyanopeptolins, Aerucyclamides, Aeruginosis and Microginins are examples of well-characterized cyanopeptides. Microcystins also share some resemblances to APs as the former possesses a ring structure, but it is comprised of 7 residues and no exocyclic amino acid. Also produced by the NRPSs apparatus, MCs bear D-amino acids, and the unusual residue (2S,3S,8S,9S)-3-amino-9-methoxy-2,6,8-trimethyl-10-phenyldeca-4,6-dienoic acid, also known as Adda, which is present in all MCs. One example of MC variant is Microcystin-LR, which is composed by (1)-Ala, (2)-Leu, (3)-N-methyl-Asp, (4)-Arg, (5)-Adda, (6)-Glu, and (7)-N-methyl-dehydro-Ala, where LR refers to 2 and 4 variable positions among MCs. It was observed in different cyanopeptides inside this class that there are different variants according to these positions, contributing to their structural diversity [10,11]. 

Cyanopeptolins are depsipeptides containing a 6-amino acid ring bearing a side chain with 1–2 residues and modified residues, such as 3-amino-6-hydroxy-2-piperidone. Cyanopeptolin A is one example of this class of cyanopeptides and is composed by (1)-fatty acid, (2)-Arg, (3)-Ahp, (4)-Leu, (5)-methyl-Phe, (6)-Val, and (7)-Thr, in this case, the β-lacton ring is formed between Arg and Thr residues and positions 2, 4, 5 and 6 are variable. Using Anabaenopeptin A as reference (Figure 2), its structure is (1)-Tyr, (2)-D-Lys, (3)-Valine, (4)-Homotyrosine, (5)N-methyl-Alanine, (6)-Phenylalanine [44]. Positions 1, 3, 4, 5, and 6 are variable concerning APs (Figure 1) and the ureido bond is formed between 1 and 2 residues. Aerucyclamides are entirely cyclic peptides, Aerucyclamide A is composed by (1)-dehydro-Thr, (2)-Gly, (3)-thiozole, (4)-Ile, (5)-dhCys, and (6)-Ile, in this case, variations were reported in positions 2, 3, 4 and 6. Different from the cyanopeptides listed until now, Aeruginosins and Microginins are linear peptides. Aeruginosin KB 676 is formed by (1)-Hpla, (2)-Ile, (3)-Choi and (4)-Arg, only position 2 presents variation with amino acid substitution, and radical changes occur in positions 3 and 4. Finally, Microginin 713 is formed by (1)-Ahda, (2)-Ala, (3)-Val, (4)-N-methyl-Tyr, and (5)-Tyr, in this case, positions 2, 3, 4, and 5 had substitutions reported [11].

Structurally, despite the major amino acid variability, Microcystins, Cyanopeptolins, and Anabaenopeptins are most similar. Microcystins and Cyanopeptolins are heptapeptides and Anabaenopeptins are hexapeptides and comparting these structures, it is possible to distinguish a ring core and a linear region. Although Microcystins are technically cyclic peptides, the Adda moiety projected outside the ring may act like the fatty acid in Cyanopeptolin A or Tyrosine in Anabaenopeptin A. The Adda moiety is crucial for MCs inhibition towards phosphatases, as its long linear chain can penetrate the enzyme active site together with other side chains, having a similar role as the exocyclic residue of APs (Tyrosine from Anabaenopeptin A), as it will be further discussed in Section 7 [11,45]. This exocyclic or even protuberant residue was not observed in Aerucyclamides that only present a cyclic structure or Aeruginosins and Microginins, which are linear structures [11]. Therefore, cyclic peptides bearing exocyclic residues and unusual and D-configuration amino acids are also found in cyanobacteria, however, the ureido linkage in cyanopeptides is, so far, an exclusive characteristic of Anabaenopeptins. 

**Figure 3:**
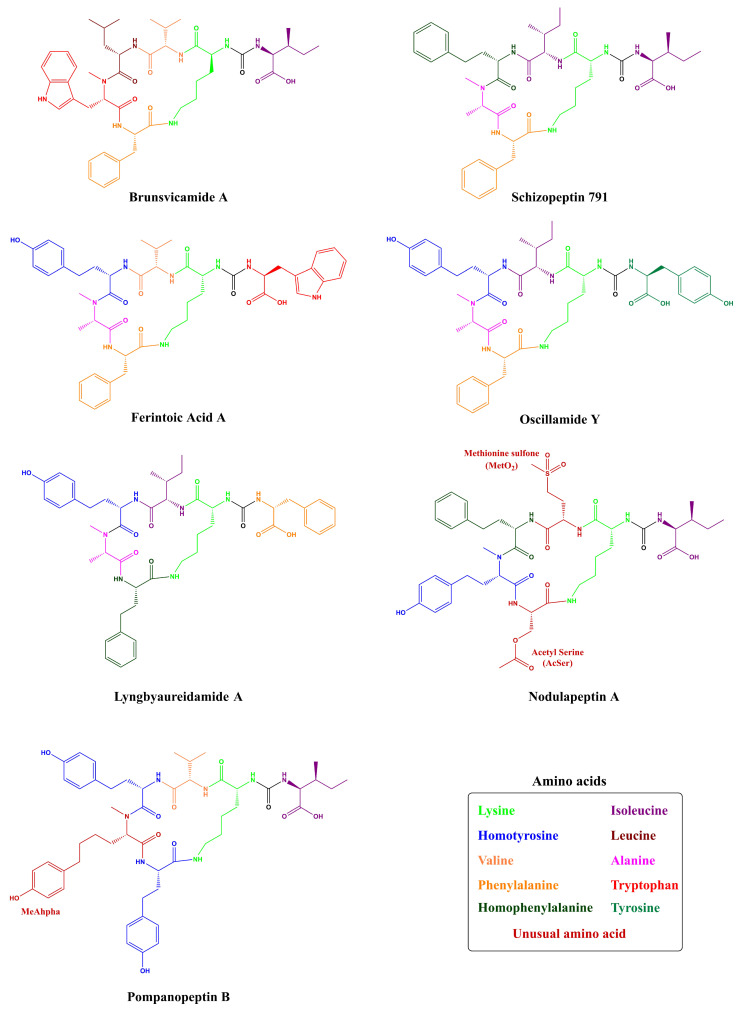
Example of different nomenclatures to anabaenopeptin-like structures [20,23,39,46,47,48,50].

All these AP variants named here so far are structurally related, differing only by amino acid substitutions responsible for their diversity (Supplementary Table S1). However, these peptides do not possess a fully systematic nomenclature, which can make it difficult to identify them as a member of a certain group of oligopeptides with similar structure. This fact is not specific to Anabaenopeptins, but cyanopeptides in general, as their denominations are frequently referring to the taxon or geographic locality from which the oligopeptide had been isolated, and also information regarding molecular weight, specific residues, or even the strain number can be used as a suffix, and some example can be seen applied to APs [11]. One example of a variant with a distinct name is the Schizopeptin 791 (Figure 3), which was named after the terrestrial cyanobacteria *Schizothrix* sp. IL-208-2-2 (Schizo-), its peptide nature (-peptin) and its molecular weight of 791 Da (791) [46]. Lyngbyaureidamides A and B are Anabaenopeptins named after their isolation from the filamentous freshwater cyanobacterium *Lyngbya* sp. SAG 36.91. These anabaenopeptin-like peptides also have an uncommon feature due to the presence of a D-Phenylalanine in the exocyclic position, being the only APs bearing an amino acid in D-configuration in this position [47]. Obtained from the marine *Lyngbya confervoides*, Pompanopeptin B is an anabaenopeptin-type peptide bearing in the fifth position the *N*-methyl-2-amino-6-(4′-hydroxyphenyl)hexanoic acid (N-Me-Ahpha), a methylated form of a residue found in Largamide C [23]. Nodulapeptins are also anabaenopeptin-like peptides and they were first identified by Fujii and co-workers [48] in the toxic *Nodularia spumigena* AV1. Among the different nomenclature of this class of cyclic hexapeptide, Nodulapeptin is one of the most used and it is often associated with the presence of Methionine (Met) or Serine (Ser) residues in position 6 of anabaenopeptin-like structures [49]. Isolated from the cyanobacteria *Tychonema* sp., Brunsvicamides A-C share a high resemblance to anabaenopeptin-like peptides obtained from sponges, thus indicating their possible cyanobacterial origin. These peptides obtained from a *Tychonema* sp. strain did not possess any homoamino acid and have a L-Lys besides D-Lys, in addition, Brunsvicamide C has an *N*-methyl-N’-formyl-D-kynurenine unit in position 5 [50].

**Figure 4:**
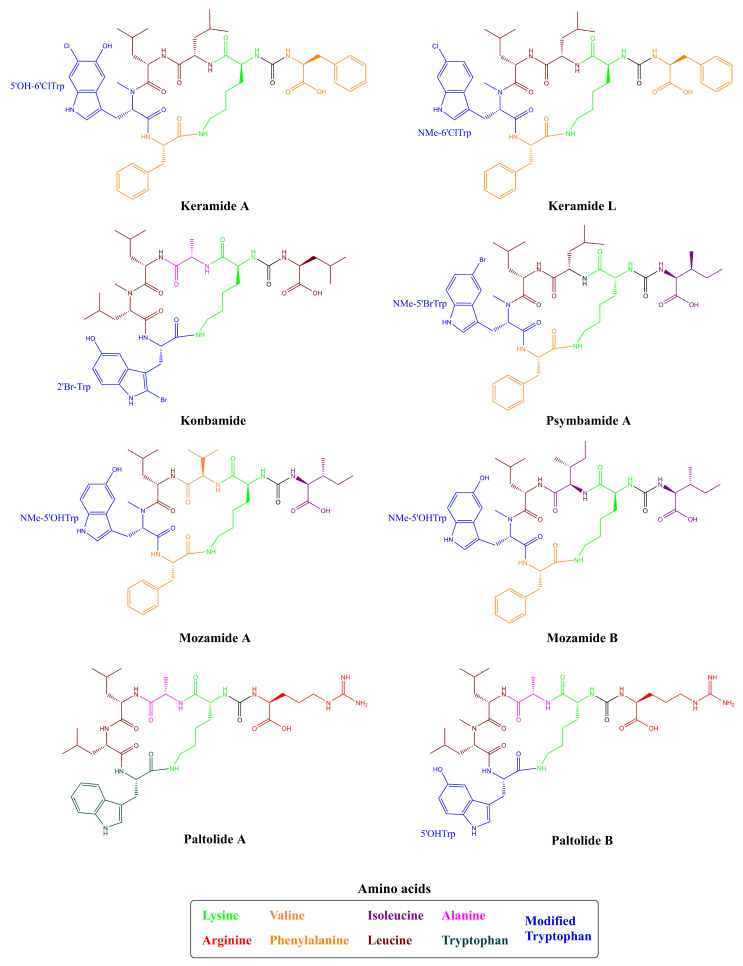
Structures of anabaenopeptin-like peptides obtained from sponges [28,29,30,31,32,33].

Besides these distinct nomenclatures and structures for Anabaenopeptins obtained from cyanobacteria, this class of peptides can also be found in sponges, which were the initial organisms to be identified the first anabaenopeptin-related compound, not in a cyanobacterium [31,32]. Konbamide and Keramide A (Table 1 and Figure 4) were isolated from the marine sponge *Theonella* sp., which showed distinct features from cyanobacterial anabaenopeptins having a cyclic hexapeptide structure and the presence of an ureido bond. Both variants have L-Lys residue and also they contain a modified Tryptophan (Trp) residue at position 6. Konbamide had 2-bromo-5-hydroxytryptophan (2’Br-Trp) in position 6; in comparison, Keramide A possessed a 6-chloro-5-hydroxy-*N*-methyltryptophan (5’OH-6’ClTrp) in position 5 [31,32]. Keramide L was detected in *Theonella* sp. SS-342 together with Keramide K (a thiazole-containing cyclic peptide not belonging to anabaenopeptin-class). Keramide L shared similar features to Konbamide and Keramide A, having a modified Trp residue in position 5: a 6-chloro-*N*-methyltryptophan (NMe-6’ClTrp) residue [30]. Besides, the marine sponge *Theonella swinhoei* demonstrated to produce a similar class of peptides, named Paltolides A-C, and 3 additional peptides which were previously detected in the Australian sponge *Melophlus* sp. [51]. Unlike Konbamide and Keramide A, Paltolides A-C and the 3 unnamed peptides have a D-Lys residue in position 2, but 4 of these structures had modified Trp residues, and 3 of these modifications were also related to the presence of a halogen element (Bromo or Chlorine) similar to Konbamide and Keramides A and L [28]. Other examples from sponges are Mozamides A and B produced by *Theonella* sp. from Mozambique. Similar to other anabaenopeptin-like peptides from sponges, both Mozamides have the same modified Trp (*N*-methyl-L-5′-hydroxytryptophan) residue in position 5 and an L-Lysine at position 2. However, both have an amino acid in D-configuration in position 3: D-valine in Mozamide A; and D-Isoleucine in Mozamide B [29].

**Figure 5:**
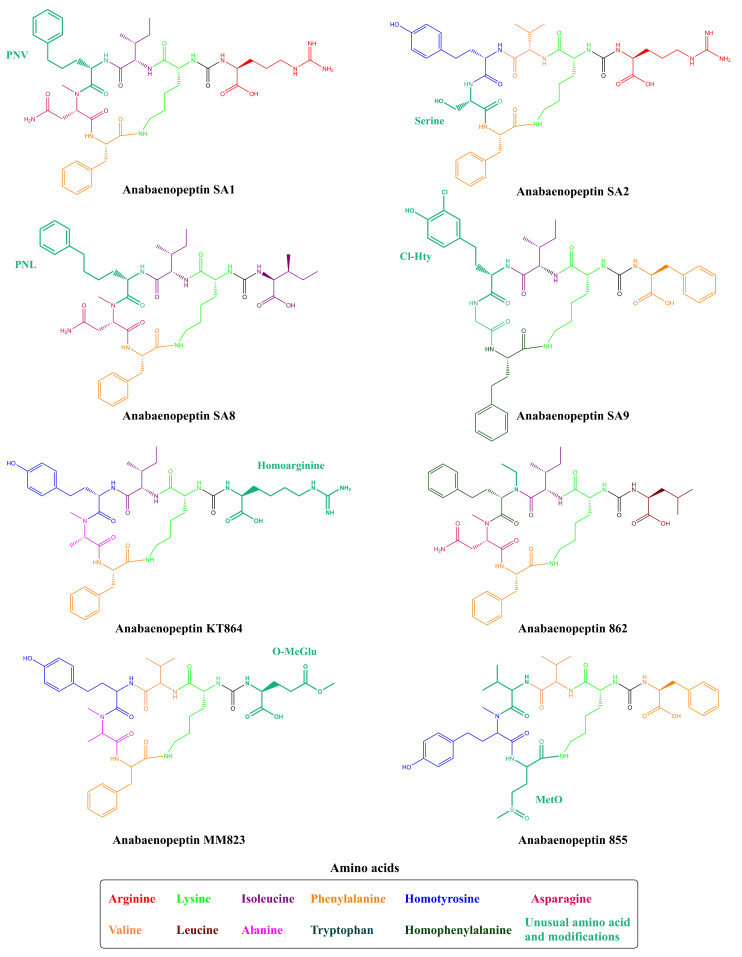
Examples of untypical features of anabaenopeptins from cyanobacteria [12,22,24,34,52,53].

To this date, 124 Anabaenopeptins variants have been identified (Supplementary Table S1 and Table 1), making it difficult to compile a systematic nomenclature and to evaluate every individual feature. To overcome this problem, several works have named new Anabaenopeptins according to their mass or even mass and specific information regarding the strain or location in which it was isolated [34]. However, some Anabaenopeptins have unusual features, typically not found in most of these peptides belonging to this class. The previously cited Ferintoic acid B has an allo-isoleucine (Allo-Ile) residue in position 6, similar to both Mozamides, which have the same residue in position 1 [29,39]. None of the Brunsvicamides have a homoamino acid and they are also the only examples of this class of peptide with L-Lys in position 2 from cyanobacteria [50]. A group of Anabaenopeptins named SA demonstrated to possess some uncommon features. Anabaenopeptin SA1, SA4, SA5, and SA7 (Figure 5) have a 5-phenylnorvaline (PNV) in position 4, which has not been previously found in any other peptide of this class. Also, Anabaenopeptin SA8 was the only one to have a 6-phenylnorleucine (PNL) residue in position 4. Additionally, APs SA2 and SA13 have a Ser residue in position 5, which has not been previously detected in the same position, but in position 6, as in Nodulapeptins [12]. Anabaenopeptins 877B, 905, 862, and 896 are some of the few examples of N-ethylated peptides [24]. The only example of homoarginine in Anabaenopeptins is from AP KT864, which has this residue in position 1 [52]. A residue of glutamate at the exocyclic position has been only described in one Anabaenopeptin: the variant MM823 [22]. Besides its common presence in position 3, Valine (Val) has been only detected in position 4 in Nodulapeptin 855C [34]. Thus, demonstrating that Anabaenopeptin peptides have huge structural diversity.

**Figure 6:**
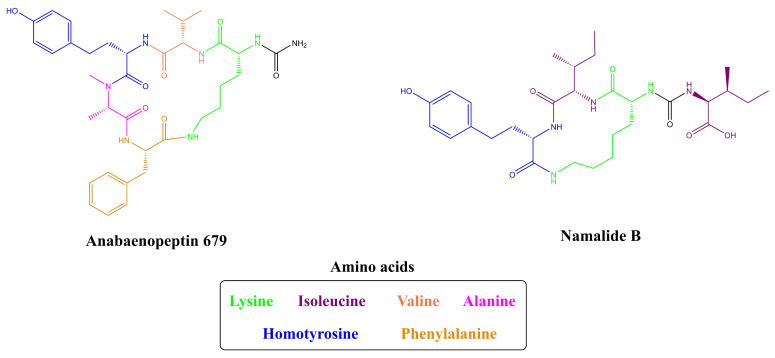
Example of Anabaenopeptins with unusual structures lacking one amino acid (Anabaenopeptin 679) and two amino acids (Namalide B) residues [53,55,56].

Unusual Anabaenopeptins lacking residues in their structures are also visualized. Anabaenopeptin 679 is the only example of an anabaenopeptin-like peptide where the exocyclic residue is absent (Figure 6). This anabaenopeptin possesses solely the ring structure, which shares the same amino acid sequence as anabaenopeptins A, B, C, D, and J [53]. Additionally, Namalides are anabaenopeptins with an atypical structure lacking two amino acids from the macrocycle. They are cyclic tetrapeptides firstly identified in the marine sponge *Siliquariaspongia mirabalis* [54] and then detected in cyanobacteria, such as *Sphaerospermopsis torques-reginae* ITEP-024 [55] and *Nostoc* sp. CENA543 [56], producing namalides B and C, and namalides B, D, E, and F, respectively. 

## 3. Occurrence of Anabaenopeptins and factors involved in their expression

Besides its great structural diversity, it appears that those peptides are usually detected in some specific genera of cyanobacteria. As can be seen in Table 2, the majority of cyanobacteria able to biosynthesize anabaenopeptins belong to genera such as *Anabaena, Microcystis, Nodularia, Oscillatoria,* and *Planktothrix*. Except for *Microcystis*, those genera are filamentous cyanobacteria belonging to the order Nostocales and Oscillatoriales. Regarding the unicellular genus, as will be discussed later (Section 4), the Anabaenopeptin NRPS cluster seems to be horizontally transferred to *Microcystis* [57]. Also, Anabaenopeptins have been detected in genera *Aphanizomenon*, *Brasilonema,* and *Desmonostoc* belonging to Nostocales order. Similar to *Oscillatoria* and *Planktothrix*, the genus *Lyngbya* belonging to Oscillatoriales demonstrated to produce anabaenopeptins. In addition, two strains of unicellular genera of cyanobacteria that belonged to Synechococcales, *Schizothrix* and *Woronichinia*, proved to have the ability to produce Anabaenopeptins. Strains belonging to filamentous cyanobacteria tend to present a higher quantity of gene clusters than the unicellular strains [58]. The heterocyst presence in some members of the order of Nostocales can also confer some advantages in the Anabaenopeptin production since this differentiated cell provides the propitious microenvironment for the nitrogen fixation, which is an element required in large amount for the production of cyanopeptides [59].

**Figure 7:**
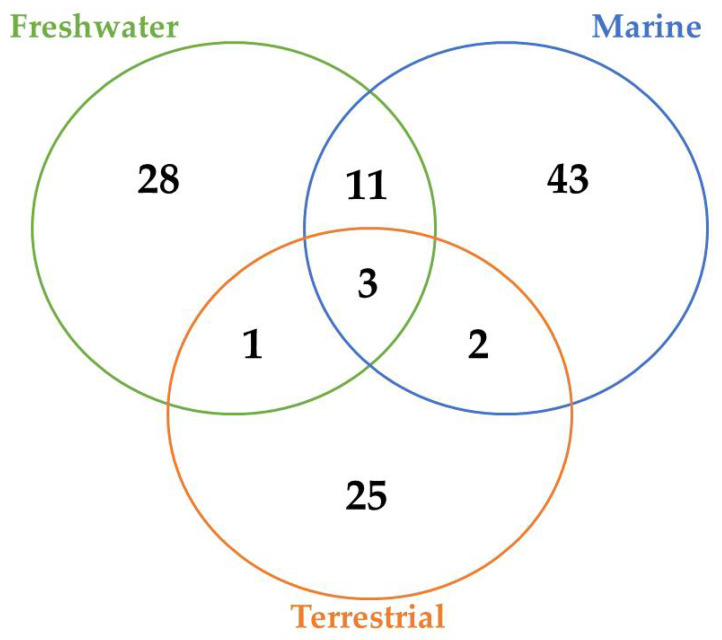
The number of Anabaenopeptins variants detected and shared among strains of cyanobacteria from different environments, including environmental samples.

**Figure 8:**
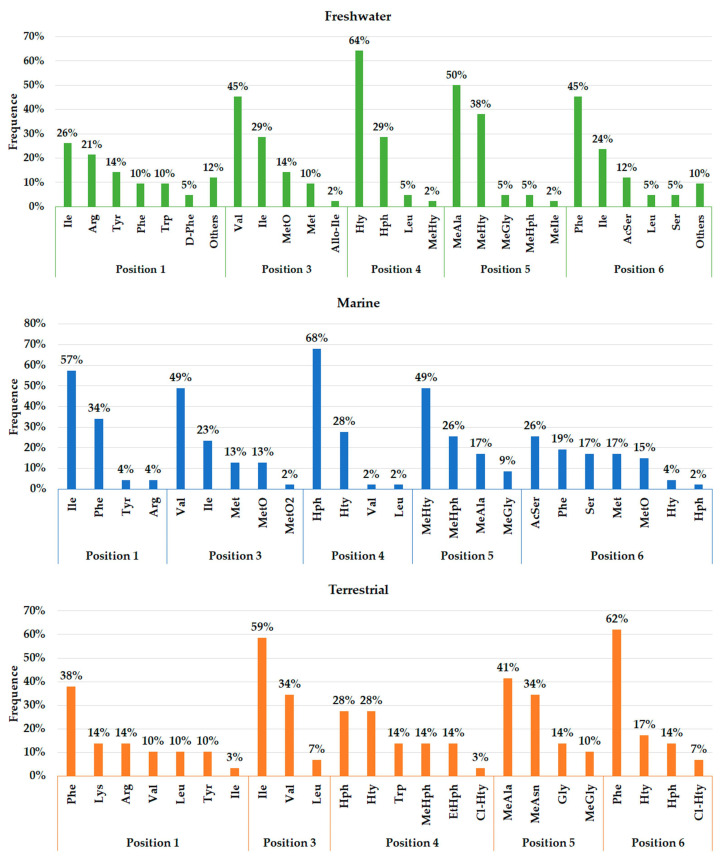
Relative frequency (%) of amino acids in positions 1 and 3–6 of variants of anabaenopeptins characterized according to their environment (freshwater, marine and terrestrial). The total number of variants with elucidated sequences were 42, 47 and 29 for freshwater, marine, and terrestrial environments, respectively. Position 2 was omitted as the D-Lys residue being conservated among AP variants.

A total of 45, 29, and 12 cyanobacteria strains from freshwater, marine and terrestrial environment have been analyzed for AP production, respectively. As seen in Table 2 and according to the literature [34,40,41,53,76,77,78,79,80,81,82,83,84,85], marine strains produced a total of 50 different variants of APs, in comparison to 43 and 34 variants from freshwater, and terrestrial strains, respectively (Figure 7). Thus, marine cyanobacteria demonstrate to produce a higher number of distinct APs variants in comparison to the remaining strains from different sources. However, APs from freshwater environments have the greatest diversity of amino acids in the majority of positions (Figure 8). Thus, these features could be associated to different obstacles faced in their respective environments as well as the fact that both belong to aquatic environments [86], however, this hypothesis requires further studies. Some of those APs are shared among different strains isolated from distinct environments: 2 anabaenopeptins (A and B) variants were detected in all ecosystems; in comparison, strains from both aquatic habitats had 13 APs variants in common (D, F, J, 807, NZ841, Oscillamide Y, and Nodulapeptins B, C, 855B, 871, 879, 897 and 915A); in contrast, only anabaenopeptin C were produced by both terrestrial and freshwater, and none Anabaenopeptin variant was shared by both terrestrial and marine strains.

According to Table 2 and Figure 7, there are AP variants shared among cyanobacteria strains from different environments according to the previous discussion. Anabaenopeptins A and B are the only variants detected in all habitats analyzed, and the only difference between those variants reside at the exocyclic residue. AP B is still the most recurrent among these oligopeptides in cyanobacteria (Table 2), corroborating with the previously raised hypothesis that this variant was the first cyanotoxin of this class to be emerged. [57]. Furthermore, the number of common anabaenopeptins variants increases when a comparison is made among strains only from aquatic habitats (freshwater and marine): Anabaenopeptins D, F, J, 807, NZ841, Oscillamide Y, and Nodulapeptins B, C, 855B, 871, 879, 897 and 915A. Besides their production by both freshwater and marine cyanobacteria, these prevalent oligopeptides seem to be more recurrent in marine environments, given that a higher number of cyanobacteria strains from this habitat are able to produce these APs comparing to freshwater, except for Oscillamide Y, which is more recurring in the latter. Among those variants, Nodulapeptin B is the most frequent in marine microorganisms. Besides, the only difference between the AP C (produced by freshwater and terrestrial strains) and both A and B variants is the exocyclic amino acid, and the former was not detected in marine cyanobacteria.

As seen in Figure 7, the environment can exert a crucial role in the biosynthesis of different APs, justifying their distribution in certain locations. The presence and frequency of certain amino acids in Anabaenopeptin structures can vary according to their respective source environment. Anabaenopeptins from both aquatic environments demonstrate to have Isoleucine as the most recurrent amino acid in position 1, while this same amino acid was detected in only one AP variant in terrestrial strains (Figure 8). Phenylalanine was highly detected in position 1 of Anabaenopeptins isolated from terrestrial strains. Then, freshwater cyanobacteria may be promising biotechnological targets due to its highest diversity of amino acids in position 1, as the exocyclic residue is crucial for its inhibitory activity [12,34,45]. Regarding the variable position 3, Anabaenopeptins from freshwater and marine environments displayed a similar pattern of amino acid frequencies, Valine (Val) being the most frequent, followed by Ile and L-Methionine sulfone (MetO2). In contrast, terrestrial strains produce several AP variants with Ile in position 3, followed by Val and Leu, the latter being absent in this position on APs detected in aquatic environments. Homotyrosine (Hty) and Homophenylalanine (Hph) are the most found residues in position 4 among APs from all habitats analyzed, however, among terrestrial and marine strains Hph is more predominantly, while Hty is commonly observed in APs from freshwater strains. Except for Glycine (Gly) in some Anabaenopeptins from terrestrial strains, all the other residues in position 5 are N-methylated. APs from non-aquatic cyanobacteria do not harbor homoamino acids in the fifth position and, in addition, Asparagine is only detected in some of those variants in the respective position. Besides their detection in position 5, homoamino acids seem to be more persistent in position 4 from those APs analyzed. Position 6 has the highest richness of amino acids among AP variants obtained from marine environments, having incorporated 7 different residues, while this position in variants from freshwater habitats have assimilated 9 different amino acids, being the second most diverse site. Such heterogeneity in the last position in APs from aquatic strains is not clear, as the first amino acid residue demonstrated to be important in Anabaenopeptin interaction towards its enzyme target [12,34,45]. This array of several amino acids detected in position 6 is not visualized in Anabaenopeptins from terrestrial strains, where Phe was the amino acid more detected, similar to those APs from freshwater microorganisms.

The identification of the external physicochemical parameters involved in the regulation of these molecules can assist in controlling and assessing their risks [87]. Furthermore, this type of information can enable a better comprehension of their functions in producing organisms [27,88,89,90,91].

Microcystins, nodularins, and saxitoxins are among the most studied toxins from cyanobacteria, in which their gene clusters can operate independently, being, therefore, able to react oppositely when exposed to the same conditions [92,93]. The relationship between the gene cluster of Anabaenopeptins with these clusters within an individual strain is not well explored, demanding more detailed studies by a holistic approach, since enabling the study of various peptides at the same time [89,90]. 

**Figure 9:**
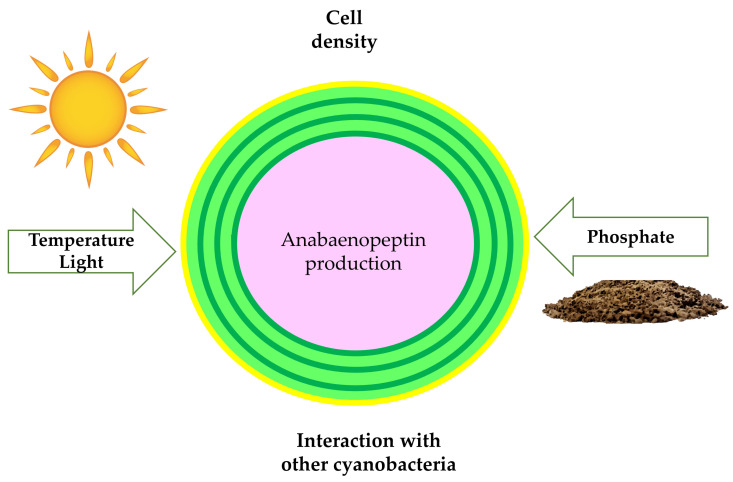
Major factors involved in anabaenopeptin regulation in cyanobacteria.

Anabaenopeptins content per cell is strongly affected by environmental factors (Figure 9). Tonk and co-workers [27] investigated the effect of light intensity, temperature, and phosphorous concentration on the growth of the cyanobacterium *Anabaena* sp. 90 as well as its production of Anabaenopeptins A and C among some MC variants and with the anabaenopeptilide 90 B. This later belongs to an underexplored group of depsipeptides, which similar to the APs has the structure of a ring with a side chain, but without the ureido linkage. In the phosphorus-limited condition, all peptides were detected in a higher amount. These data match the result of Teikari and colleagues [94], who studied the same *Anabaena* strain and encountered higher quantities of transcripts belonged to anabaenopeptins, anabaenopeptilide, and microcystins gene clusters under low-phosphate conditions. Phosphate limitation also increases the content of protease inhibitors of the cyanobacterium *M. aeruginosa* NIVA CYA 43 [95].

In contrast, anabaenopeptilide 90B responded in a different way of Anabaenopeptins A and C to light exposure. The former increases considerably with light intensity while the others had their production reduced. These two peptide groups exhibit a compensatory dynamic, where the reduction of one is accompanied by the increase of the other. This strategy employed by cyanobacteria ensures the constitutive production of peptides with similar functions in an unstable environment, increasing its survival change. For example, anabaenopeptilides are also serine protease inhibitors, having near functions to APs in the cell. In respect to the temperature, its increase favored anabaenopeptins production but resulted in a slower growth rate [27]. Such observations demonstrate that APs production is a constitutive process and is not always positively related to the growth of *Anabaena* sp. 90, contrasting, therefore, with the argument that the best growth conditions are more favorable to toxins production. 

A compensatory mechanism has been described by other authors in cyanobacteria [88,89,90,96]. A comparison of the oligopeptide profile of *M. aeruginosa* PCC 7806 and its microcystin-deficient mutant revealed that the loss of this toxin has as consequence the increased production of cyanopeptolins and aeruginosins [90]. Pereira and co-workers [96] observed similar behavior for different variants of MCs in *Radiocystis fernandoii* 28. Microcystin-RR exhibited an opposite response to those observed for microcystins YR, FR, and WR under distinct light conditions in this strain. This type of modification can strongly affect its final toxicity since MC variants display different toxicity degrees. Another possible alteration consists of a change of antifeedant potential. APs significantly vary as substrate specificity, exhibiting different bioactivity. In a previous study, the inactivation of the genes involved in the synthesis of anabaenopeptilides in *Anabaena* sp. 90 resulted in a considerable increase in the production of anabaenopeptins [88]. One plausible explanation for this phenomenon is that the knockout of genes involved in the biosynthesis of some oligopeptides can lead to a credit of energy, which is allocated to the production of the remaining peptides [90].

The impact of the cell density on anabaenopeptin production and other oligopeptides have been investigated since it has been related to the increase in the production of some antibiotics. Moreover, this phenomenon can provide valuable data about probable alterations during bloom processes and mat formation [97,98]. High-density cultivation of photosynthetic microorganisms can be a challenge as the light availability decreases with cell density. Guljamow and co-workers [21] utilized a two-tier vessel developed by Bähr and co-workers [99] to cultivate *N. punctiforme* PCC 73102 and *Nostoc* sp. strain KVJ2 at high cell density and observed a higher diversity of secondary metabolites. Anabaenopeptin was absent in *Nostoc* sp. strain KVJ2 biomass obtained under conventional cultivation. High-density cultivation of this strain revealed the presence of three novel anabaenopeptins (KVJ827, KVJ841, and KVJ811). The increase of the content of these APs in the strain KVJ2 is attributed to a higher number of transcriptions among the cells. In the conventional cultivation, the distribution of the *aptA* transcripts (an NRPS gene related to AP production) was restricted only to a cell at (pre-)akinete state while in high-cell density culture, this transcript was widely distributed among the vegetative cells [21].

The interaction between different chemotypes of cyanobacteria in a water body can provoke significant alterations in their secondary metabolites profile. Consequently, differences are observed between laboratory culture and natural environments. In co-culture with *M. aeruginosa* PCC 7806, the non-microcystin-producing strain *M. aeruginosa* PCC 9432 enhanced its bioactive peptide content, including Ferintoic acids A and B [90]. These findings suggest the release of diffusible signals by cyanobacteria with the capacity of regulating the production of APs. The chemical nature of such metabolites was not determined in this study. However, certain oligopeptides can fill the signaling function since they are occasionally found in the extracellular compartment, acting as infochemicals. In addition to peptides, cyanobacterial exudate has also some nutrients, which affect the production of certain toxins and can be, consequently, responsible for the increase of Ferintoic acids A and B in *M. aeruginosa* PCC 9432 [100]. In a later study, the supplementation of the culture medium of a *P. agardhii* with two oligopeptides extracts from samples of *P. agardhii* as the predominant cyanobacterial species had different effects on the synthesis of the peptides of this strain. Both extracts showed a positive impact on biomass accumulation and chlorophyll-a production, being attributed to those nutrients and oligopeptides now present. The high nutritional content of the extracts is associated with the ability of cyanobacterial in fixing nitrogen and producing vitamins, phytohormones, and polysaccharides. Three out of four anabaenopeptins maintained constant (m/z 851, 844, and 837) while the variant with m/z 828 was substituted by other with m/z 923. One of the extracts increased the anabaenopeptin content of variants m/z 844, 851, and 837 while the other diminished the quantity of these last two [101]. The opposite responses to these extracts may be assigned to the content differences observed between them. The extract responsible for reducing the APs expression exhibits a superior concentration of nitrate and phosphate, which, as was previously mentioned has a negative effect on the production of anabaenopeptin [27]. 

In addition to interaction with other cyanobacteria, these microorganisms are capable to establish symbiotic associations with invertebrates, such as corals, mollusks, and sponges. Both organisms can be benefited during this consortium through secondary metabolite production, for example [102]. Sponges host an enormous quantity of microorganisms belonging to diverse phyla, where cyanobacteria are mainly represented by genera *Aphanocapsa*, *Synechocystis*, *Phormidium*, and *Oscillatoria* [103]. These photosynthetic microorganisms can occupy either extra- or intracellular spaces, aiding the host in the control of the redox potential, supplying pigments and energy through carbon fixation, and in the defense mechanism by the production of secondary metabolites. Published reports have demonstrated that as a consequence of these processes, cyanobacteria have their metabolic profile altered, resulting in the production of distinct variants of natural products. The compound 2-(2’,4’-dibromophenyl)-4,6-dibromophenol is solely biosynthesized by a cyanobacterium belonging to genus *Oscillatoria* in association with the sponge *Dysidea herbacea* [104]. These factors corroborate with the hypothesis that anabaenopeptins primarily observed in sponges could be of cyanobacterial origin, as brominated APs variants were isolated only from sponges [28,31,33] and the *Oscillatoria* genus is known for APs production. For instance, the polyketide nosperin and some variants of oligopeptide nostopeptolide are encountered exclusively during symbiosis, which may be the same mechanism for anabaenopeptin variants production found in sponges.

## 4. Biosynthesis

The features of Anabaenopeptins are related to Non-Ribosomal Peptide Synthetases (NRPSs), which operate with a nucleic acid-free mechanism at the protein level and are structured as multifunctional proteins. NRPSs are organized as gene clusters in bacteria, usually possessing all the proteins required for proper biosynthesis of the secondary metabolites, from the generation of building blocks to product transport [105,106,107].

The variability of NRP structures, both cyclic and linear, reflects the concept of the complex modular system of NRPSs organized as an assembly line. Each module is responsible for the activation and coupling of an amino acid to the respective oligopeptide being synthesized. The principle known as the collinearity rule dictates that, for example, a hexapeptide requires six modules to be produced. Those modules are composed of enzymatic domains present in an NRPS, which are responsible for specific biosynthetic steps, as amino acid activation, bond formation, and oligopeptide liberation. Besides the initiation module, an elongation module from an NRPS requires, at least, an Adenylation-domain (A-domain) for amino acid recognition and activation; the Thiolation-domain (T-domain), required to carry the synthesized peptide; and a Condensation-domain (C-domain), responsible for the peptide bond formation. The last module of this assembly line requires the Thioesterase-domain (Te-domain) for the proper maturation of the peptide, also responsible for the cyclization step [18,105,106,107,108].

Similar to other peptides produced by NRPS, the biosynthesis of APs requires all the specific steps of the assembly line. Besides, due to some specific characteristics present in this cyclic hexapeptide and its variants, other proteins and domains can also be related to its synthesis, as the biosynthetic apparatus for homoamino acid production and domains for D-Lys formation (Epimerization-domain; E-domain) and N-methylation of specific residues (Methylation-domain; M-domain) [18,19,105,106,108,109].

Besides the fact that the anabaenopeptin structure’s first detection in cyanobacteria occurred in 1995 [20], its gene cluster was only described ten years later in a *Planktothrix rubescens* strain [18]. The gene cluster detected in this cyanobacterium comprised of 5 genes (*ana*ABCDE): 4 NRPSs, and an ATP-Binding Cassette-transporter (ABC-transporter) protein. It was also visualized NRPSs possessing an epimerase domain (AnaA) and a methyltransferase domain (AnaC), which could be related to typical features encountered in APs, such as D-Lys and N-methylated amino acids, respectively. Only one cluster was detected in this organism, and it was attributed for the biosynthesis of all four peptides produced: Anabaenopeptin A, B, F, and Oscillamide Y, which differ by the combinatory of two residues in two distinct positions: (Tyr/Arg)-Lys-(Val/Ile)-Hty-MeAla-Phe. Thus, this phenomenon indicates that these NRPSs demonstrated a certain degree of promiscuity regarding their substrates and A-domains, as different amino acids can interact with the same catalytic site [18].

Rouhiainen and co-workers [110] detected gene clusters related to the production of APs in *Anabaena* sp. 90, *Nodularia spumigena* CCY9414, and *Nostoc punctiforme* PCC3102. In *Anabaena* sp. 90, five Open Reading Frame (ORF) were identified to be encoding NRPSs (*aptA1, aptA2, aptB, aptC,* and *aptD)* and two additional genes to be encoding proteins with similarity to HMGL-family (*aptE*) and ABC-transporter protein (*aptF*). When compared to the clusters identified in *N. spumigena* and *N. punctiforme,* 4 NRPS and two homolog proteins to AptE and -F were also detected, indicating that *Anabaena* sp. had an additional NRPS gene (*aptA1* and *aptA2*). Similar to AnaA from *Planktothrix rubescens* NIVA-CYA 98, AptA1 and AptA2 also have an epimerase domain indicating their role as initial enzymes, and AptC possessing the N-methyltransferase domain as AnaC [110]. The proteins AptA1/AptA2, AptB, AptC, and AptD are homologs to the NRPS proteins AnaA, AnaB, AnaC, and AnaD, sharing the same functions, respectively.

A genomic analysis of *Sphaerospermopsis torques-reginae* ITEP-024 accomplished by Lima and colleagues [107] demonstrated that the *apt* gene cluster is close to the spumigin cluster. Both AP and spumigin are peptides with protease inhibitory activity which usually possess Homophenylalanine and Homotyrosine residues, then indicating that both NRPS apparatus share a biosynthetic cluster related to the production of these nonproteinogenic residues. The *apt* gene cluster of *S. torques-reginae* strain has a similar organization to the anabaenopeptin clusters from *Anabaena, Nodularia, Nostoc,* and *Plaktothrix* [18,110]. Thus, its cluster also holds four genes encoding a six-module NRPS (*apt*ABCD), where the Te-domain is present at the last module, then being responsible for the final step of AP production, similarly to other NRPS products [107].

Entfellner and co-workers [57] suggested that the AP cluster could be transferred among cyanobacterial species due to horizontal gene transfer (HGT). This hypothesis is supported by the high similarity visualized between the *apnA-E* cluster from *Planktothrix* and *Microcystis* composed by *apnA*, *apnB, apnC, apnD* and *apnE*, which genes codified proteins homologs to AnaA/AptA, AnaB/AptB, AnaC/AptC, AnaD/AptD, and AnaE/AptF, respectively. Some strains belonging to the *Planktothrix* genus demonstrated to possess the same AP cluster, but not all of them, thus suggesting that the common ancestors of these organisms did not have the NRPS apparatus for AP biosynthesis, which could be visualized by a phylogenetic analysis using *apnA-E* clusters as biological markers. By phylogenetic analysis of different sequences of anabaenopeptin cluster, it could be inferred that an ancestral cluster was introduced into the chromosome of a *Planktothrix* strain and diversified into different variants, which could be grouped according to *apnA* sequences. Thus, the high frequency of AP producers belonging to *Microcystis* and *Planktothrix* in nature could be an indication of this mechanism of genetic transference by the AP cluster and its wide distribution among those genera, requiring further analysis of the same mechanism in other AP producers, such as *Anabaena, Aphanizomenon, Nodularia, Nostoc, Oscillatoria,* and *Lyngbya* (Table 2) [57].

It had been detected in *Nostoc* sp. CENA543 six variants of APs. Through genomic analysis, a gene cluster of 26 kb containing four NRPS and additional enzymes was visualized related to AP production. Following the same pattern, the NRPS proteins were AptABCD, and the additional enzymes were an ABC-transporter, 2-isopropylmalate synthase (HphA), and an ORF similar to Nuclear Transport Factor-2 (NFT2) proteins [56].

**Figure 10:**
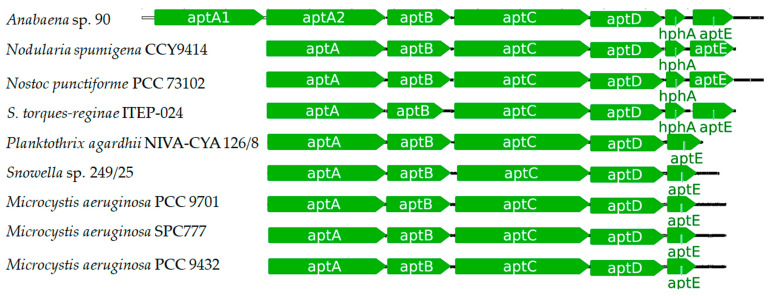
Anabaenopeptin cluster (*apt*) organization from different cyanobacteria strains. The genes *aptA1*, *aptA2*, *aptA*, *aptB*, *aptC*, *aptD* and *aptE* are Non-Ribosomal Peptide Synthetases (NRPSs) related to Anabaenopeptin production; *hphA* gene belongs to homoamino acid biosynthetic pathway and *hph*ABCD cluster. These clusters were obtained according to their accession codes (AC) from National Center for Biotechnology Information (NCBI): *Anabaena* sp. 90 (AC: GU174493), *Nodularia spumigena* CCY9414 (AC: CP007203), *Nostoc punctiforme* PCC 73102 (AC: CP001037), *Sphaerospermopsis torques-reginae* ITEP-024 (AC: KX788858), *Planktothrix agardhii* NIVA-CYA 126/8 (AC: EF672686), *Snowella* sp. 249/25 (AC: MF741700), *Microcystis aeruginosa* PCC 9701 (AC: HE974200), *Microcystis aeruginosa* SPC777 (AC: PRJNA205171), *Microcystis aeruginosa* PCC 9432 (AC: HE972547). This information is available on the public database NCBI (https://www.ncbi.nlm.nih.gov/; accessed on 16 March 2021).

**Figure 11:**
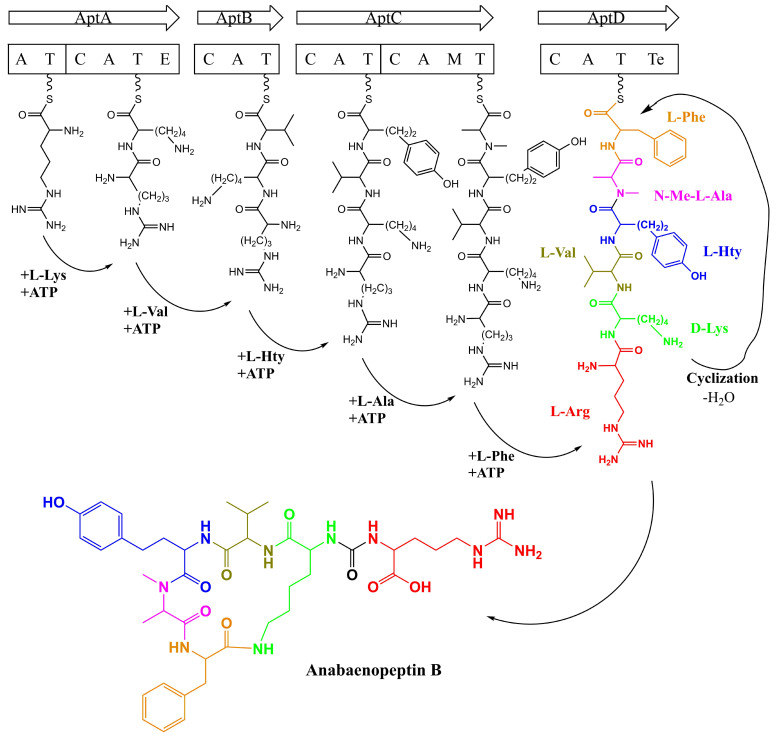
Scheme of biosynthesis of anabaenopeptin B in *Anabaena* sp. 90 by NRPS apparatus [107,110]. A: adenylation domain; T: thiolation domain; C: condensation domain; E: epimerization domain; M: N-methylation domain; Te: thioesterase domain.

Thus, as discussed, several AP clusters have been identified (Figure 10) and their nomenclatures are not standardized, which usually are assigned according to the strains detected. For example: *ana* and *apn* for *Planktothrix* [18,57,111,112]; *apt* for *Anabaena, Microcystis, Nodularia, Nostoc* and *Sphaerospermosis* [56,107,110]; and even *kon* from *Candidatus Entotheonella* sp. TSY referencing the konbamide biosynthetic gene cluster [113]. Among these nomenclatures, *apt* is the most recurring, being applied to refer the AP gene cluster along this manuscript. However, all anabaenopeptin gene clusters from these different strains of cyanobacteria share common features. The first NRPS, AptA, is a bimodular initiation enzyme containing two A-domains, two T-domains, one C-domain, and one E-domain. The second NRPS enzyme, AptB, contains one elongation module (condensation, adenylation, and thiolation domains), followed by the third enzyme, AptC, which is an NRPS enzyme with two elongation modules, which commonly contains distinct domains related to peptide modification, such as N-methyltransferases. Finally, the termination module from AptD comprises an elongation module which also includes a Te-domain (Figure 10 and Figure 11). Then, it totalizes 6 modules, following the collinearity rule and confirmed by bioinformatic analyses regarding the specificity of each module with its amino acid [18,56,57,107,110,111,112].

The first adenylation domain from the NRPS apparatus belonging to the first module of AptA (Figure 10 and Figure 11) had been analyzed by several works due to the inhibitory role of the first amino acid residue towards specific enzymes [57,111]. Evolutionary analysis coupled to molecular biology demonstrated that one of the first anabaenopeptin to be produced possessed Arg at position 1, such as anabaenopeptin B (Figure 2). This data corroborates with the inhibitory activity of carboxypeptidase B of AP variants bearing Arg at the exocyclic position, which is greater than Tyr, Phe, and Ile. In addition, analysis of *Planktothrix* producers strains demonstrated a high frequency of AP B producers (83 out of 89 strains), followed by AP A, AP F, and Oscillamide Y (55%, 45%, and 33% of the strains), corroborating with Table 2 [57]. Some wild-type adenylation domains from the first module of AptA demonstrated to be highly specific for arginine and tyrosine, and single point mutations within this domain can result in significant substrate promiscuity [57,110,111,112]. Due to its high frequency, inhibition towards carboxypeptidase B, and the possibility to be the first oligopeptide of its group to be originated, the biosynthesis of Anabaenopeptin B is outlined in Figure 11 and will be used as a standard for APs production.

Through a search of APs biosynthetic clusters in several cyanobacteria, Shishido and colleagues [56] detected that the majority of strains of cyanobacteria contained only one *apt*A gene. However, ten cyanobacteria and the tectomicrobia *Candidatus Entotheonella* sp. TSY1 possessed two alternative *apt*A genes. Thus, under other works [18,56,57,107,110,111,113], the biosynthesis initiation of APs has two different approaches. The first one is the NRPS with the presence of two starter modules with distinct substrate specificities that can produce different variants of APs. The second mechanism is due to the promiscuity of the first adenylation domain of AptA, producing different variants at position one [112]. Both mechanisms can increase the chemical diversity of Anabaenopeptins produced.

As discussed previously, Rouhiainen and co-workers [110] identified an anabaenopeptin cluster from *Anabaena* sp. 90, possessing one additional NRPS enzyme with two modules (AptA1 and/or AptA2). This cyanobacterium was able to produce 3 different AP variants differing at position one. Through sequence comparison and substrate specificity analysis, it had been demonstrated that the first adenylation domain of AptA1 had an affinity to L-Lys and L-Arginine (Arg), while AptA2 demonstrated to interact with L-Tyr. Both adenylation domains from the second module of AptA1 and AptA2 incorporated D-Lys. Thus, demonstrating that *Anabaena* sp. 90 carried two distinct initiations NRPS producing different variants of anabaenopeptin, which a similar mechanism could also be visualized for puwainaphycins and minutissamides [110,114]. However, in Figure 11, only AptA1 is represented due to its specificity towards Arg.

Regarding the promiscuity of the adenylation domains aiming to understand the production of distinct AP variants, the adenylation domain of AptA from *Plaktothrix agardhii* PCC 7821 had been evaluated and concluded that it demonstrated to be bispecific for two different amino acids: Arg and Tyr. This feature corroborates with the variants produced by this strain of *P*. *agardhii*: Anabaenopeptins 908A and 915, which differs solely in the exocyclic residue (Arg or Tyr) [111,112]. A similar pattern had been visualized in *Planktothrix rubescens* NIVA-CYA 98, which possesses only one AP cluster, but it was able to biosynthesize different variants of anabaenopeptin differing at the exocyclic position (Tyr and Arg) and the third position (Val and Ile) [18].

One important feature encountered only in Anabaenopeptin among cyanobacterial peptides is the ureido linkage between the first and second residues [34,49]. However, this linkage can also be found in other natural products, including pacidomycins, mureidomycins, napsmycins, and syringolin A. This configuration is not common due to the mechanism present in NRPSs, which assembles amide bonds in an approach where the chain polarity remains unidirectional. The presence of ureido linkage alters this polarity due to the presence of N-to-N terminal condensation. Then, a specific enzyme and/or domain must be present in NRPSs involved at the ureido linkage formation, suggesting a possible role of the first elongation module in their formation [115].

When comparing the initial NRPSs genes encoding both modules of AptA from *Anabaena* sp. 90, *Nodularia spumigena* CCY9414, and *Nostoc punctiforme* PCC 73102, they all contained typical adenylation and condensation domains, also demonstrating highly conserved motifs. Besides their conservation, one hypothesis was that both modules of initiation and elongation of AptA would be related to ureido bond formation, similar to the SylC protein, from *Pseudomonas syringae*, which role is the catalysis of the ureido linkage between two Val residues from syringolin A [110,115]. Though the SylC protein possesses a domain with structural similarity to acetyltransferase between the A- and C-domains from the NRPSs, which is responsible for the ureido linkage formation and no homologous is present in the anabaenopeptins synthetases, suggesting a different mechanism for this step during AP biosynthesis [107,115].

Besides the initiation step and the formation of the ureido bond between the first residue and the conserved Lys, several steps of elongation of the peptide are required to produce a fully mature peptide. The signature sequences analyzed of the A-domains of these NRPS enzymes, such as AptB and AptC, are consistent with the respective amino acid residue of the final product and confirmed in vitro by biochemical methods. Also, usually, the fifth module bears an *N*-methyltransferase domain, as seen in AptC and their homologs, responsible for the *N*-methyl in Ala in position 5 of Anabaenopeptin B, as seen in Figure 11 [110].

Unlike the initiation enzyme related to residues at position 1 and 2, clusters related to AP production has not been shown to possess more than one NRPS for each residue. Thus, the variants produced by the cyanobacterial differing at positions 3–6 are biosynthesized due to the promiscuity of the adenylation domains of AptBCD. This phenomenon can be visualized by innumerous AP variants differing at those positions with only one correspondent gene cluster in the genome, for example, *Nostoc* sp. CENA 543 producing six variants [56]. 

Anabaenopeptins usually have homoamino acids at positions 4 and 5, which are added by AptC during elongation steps, as visualized in Figure 11 by the additional Hty added in position 4. The AptE, now known as HphA, was first suggested to be responsible for ureido linkage formation and is related to homoamino acid synthesis [110]. Succeeding previous works, it has been elucidated that AptE belongs to a biosynthetic cluster named *hphABCD*. Genes from *hph* cluster are frequently detected in the same genomic region as *apt* and *spu* clusters, which both products, Anabaenopeptins and Spumigins, are peptides displaying protease inhibitory activity and homoamino acids. A genomic analysis of *Sphaerospermopsis torques-reginae* ITEP-024 demonstrated that both Spumigin and Anabaenopeptin clusters were present in proximity in the genome. In between both clusters, the *hphABCD* biosynthetic cluster and additional genes were detected in this region, which a similar organization was also visualized in *Nodularia spumigena* CCY9414 [107]. The *hph* genes are responsible for the biosynthesis of Hph and Hty, nonproteinogenic amino acids commonly found in both anabaenopeptin and spumigin [116]. Thus, indicating that HphA is not responsible for ureido linkage formation but behind the supply of both Hph and Hty. In addition, the presence of the homophenylalanine and homotyrosine biosynthetic enzymes in this region could suggest that this cluster is supplying both homoamino acids for APs and Spumigins [107]. In accordance with Lima and co-workers [107], Shishido and colleagues [56] also visualized that from 56 genomes analyzed containing the *apt* cluster all demonstrated to possess the *hph* biosynthetic cluster, except for *Scytonema hofmanii* PCC 7110 and *Candidatus Entotheonella* sp. TSY. The genes encoding the proteins HphABCD were frequently found upstream or downstream of the AP cluster, supporting the hypothesis about their roles in providing homoamino acids to APs [107].

Thus, homoamino acids are produced by the HphABCD enzymes and then incorporated by the NRPS apparatus. In addition, these non-proteinogenic amino acids can also be further modified by the NRPS enzymes, considering that residues at position 5 are mostly methylated by the N-methylation domain in the second module of AptC. However, methylation of residues at position 4 was also visualized, such in Ferintoic acids A and B [39], Anabaenopeptin E [37], 863, 891, 848, and 882 [24].

The final step for Anabaenopeptin production is mediated by a Te-domain, which is commonly associated with the termination process of the biosynthesis of NRPS peptides. Thus, after the incorporation of the last residue, for example, L-Phenylalanine in AP B (Figure 11), these domains can be involved with the release of the peptide by hydrolysis, or even cyclization involving peptidic or ester bonds [19,106]. The last NRPS enzymes AptD and its homologs [18,111] bear the thioesterase domain, suggesting then their role as the termination step.

Besides those typical alterations to the amino acid residues discussed, several variants of APs have been found with different modifications, such as ethylated (Figure 2, Figure 3, and Figure 5), acetylated, and oxidized residues [22,24,34]. In addition to such modifications during the elongation steps by the NRPS, an analysis of cytochrome P450 monooxygenases from cyanobacteria revealed that some proteins of this class may be related to anabaenopeptin modifications. In *Synechococcus* sp. PCC 7502, it had been suggested that a P450 belonging to CYP110 is involved in the production of Anabaenopeptin NZ857. *Anabaena* sp. TAU NZ-3-1 was capable to coproduce this anabaenopeptin and APs NZ 825 and NZ841. Anabaenopeptin NZ857 differs from AP NZ825 and AP NZ841 by the number of oxidized residues at positions 4 and 6. Anabaenopeptin NZ857 has in both positions 4 and 6 the homotyrosine residue, while the other peptides have at least one homophenylalanine. Besides the possible relation of cytochrome P450 in anabaenopeptin production, its possible catalytic role has not been demonstrated [117].

Regarding the unusual anabaenopeptins lacking residues in their structure, the biosynthesis of Anabaenopeptin 679 (Figure 6) has not been described so far [53], requiring further analysis of its production. Due to Namalide similarity to APs, it has been suggested that the biosynthesis of this tetrapeptide is realized by the *apt* cluster, as during a genomic screening of both namalides-producing cyanobacteria no exclusive cluster related to the production of these peptides have been found. The prediction of amino acids incorporation of adenylation domains of AptABCD is in accordance with both AP and Namalides. Thus, the preliminary results obtained by Shishido and co-workers [56] strongly suggested that Namalides are biosynthesized by *apt* cluster through a module skipping event. During synthesis, the second domain of AptC and the C-domain of AptD (but not the thioesterase domain) are ignored resulting in the production of namalides, similar to the module-skipping process of Myxochromide from myxobacteria [56].

## 5. Ecology

**Figure 12:**
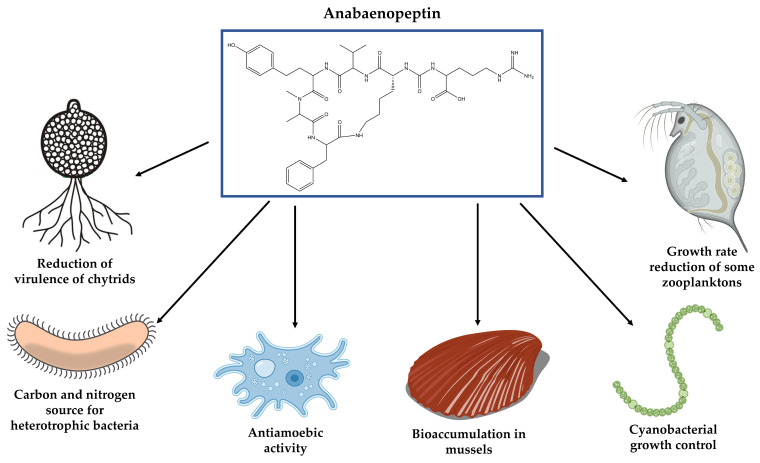
Ecological relevance of anabaenopeptins.

Cyanopeptides confer a competitive advantage for their producing organisms due to their toxicity, which effect has been examined against parasites and grazers (Figure 12) [118,119]. Other strategies, such as colony formation and filaments aggregation with low nutrition content have also been documented as a defensive mechanism [120]. However, they cannot, on some occasions, be sufficient to explain the different susceptibility levels encountered among cyanobacterial populations [121]. 

Anabaenopeptin presence in the cyanobacterial extract can confer a certain level of protection against some predators but is not a determining factor in the process as illustrated by the work developed by Urrutia-Cordero and coworkers [122]. These authors attested anti-amoeba activity against *Acanthamoeba castellanii* by *Microcystis* strains capable of producing either APs or MCs. Among the tested strains, the anabaenopeptin-producing was the one that caused the highest mortality rate. In contrast, the existence of the same APs in the extract of *A. lemmermannii* NIVA-CYA 426 did not result in any type of activity for the protozoan. Due to APs and MCs inhibitory activities against phosphatase, the loss of cytoskeleton integrity of *A. castellanii* was associated with the action of these cyanopeptides, which led to impairment of crucial functions associated with cytoplasmic projections, including motility and feeding.

Deleterious effects in organisms belonging to aquatic fauna were also linked to APs production and other cyanopeptides [123,124,125]. The negative impact of these metabolites can partially justify the substitution of large-bodied zooplankton by small-bodied species during the blooming process since they affect differently these living beings [126,127]. The absorption of such molecules can occur by ingestion of cyanobacteria or through uptake of water. Like the filtration system of large-bodied zooplankton has a greater tendency to absorb these microorganisms, they are more susceptible to the effect of toxins [128].

Some published reports have focused solely on the effect on a determined organism by an individual oligopeptide, especially MCs [92,129]. However, this type of investigation is not sufficient to verify the real impact of cyanobacterial bloom in the environment. Studies that employ only one type of solvent to obtain the cyanobacterial extract can also provide limited information since these microorganisms harbor an enormous variety of metabolites with distinct polarity, which are not, therefore, totally isolated and investigated during this type of analyses. Even though APs concentration in the aquatic environment can exceed 1 g.L^−1^, they pose unknown consequences for human health [15]. Furthermore, their full effects on other animals are largely unknown. In Zebrafish (*Danio rerio*), an animal model very close to the human being, APs B and F as well as Oscillamide Y do not have any significant effect on the mortality of their embryos [130]. Otherwise, another study demonstrated that the APs A, B, and F exhibited the greatest toxicity as compared to other cyanopeptides, such as microcystin-RR, microginin 690, and cyanopeptolins CYP-1007, CYP-1020, and CYP-1041 to the nematode *Caenorhabditis elegans*. The exposure to these understudied toxins was responsible for diminishing the reproduction potential of this worm, affecting the brood size, the hatching time of eggs and vulvar integrity. Moreover, lifespan was also reduced by nearly 5 days [131]. 

Concerning APs action in other animals, Pawlik-Skowrońska and colleagues [127] demonstrated that extracts containing Anabaenopeptins originated from bloom samples, where the predominant species were *P. agardhii* or *Microcystis* sp., caused different responses in the behavior of planktonic species *Daphnia pulex* and *Brachionus calyciflorus*. The unicellular cyanobacterium extract did not cause acute toxicity to any of the investigated zooplanktons. Otherwise, the *Planktothrix* extract strongly reduced the survivorship rate of *D. pulex*. This difference was attributed to the oligopeptide profile in the extract, which considerably varies as to their quantity and structure. Anabaenopeptin and Aeruginoside had a superior contribution in *P. agardhii* extract as compared to *Microcystis* one, suggesting that those oligopeptides act in synergism. A similar analysis harboring a larger number of organisms, verified the toxicity of *Nodularia spumigena* extracts against the crustaceans *Thamnocephalus platyurus* and *Artemia franciscana* and also the bioaccumulation of their oligopeptides in these invertebrates and some blue mussels [118]. Nine APs and nodularin were encountered in the mussels collected from a bloom formed by this cyanobacterium. In *T. platyurus and A. franciscana*, the exposure to *Nodularia spumigena* extract results in the accumulation of various Anabaenopeptins, one aeruginosin, and one spumigin. The cyclic structure of APs confers them chemical stability preventing their degradation by the mussels tested, as linear peptides were not detected. Moreover, it also led to an increased mortality rate for both organisms. Among the fractions obtained from *N. spumigena* biomass extract, that with APs and a demethylated form of nodularin exerted the highest acute toxicity effect.

Anabaenopeptins also participate in the defensive mechanism of *Planktothrix* allowing, therefore, its dominance towards pathogens in the same environment. A comparison between a wild-type strain of *P. agardhii* NIVA CYA 126/8 and their mutants with dysfunctions in the production of APs, microviridins or MCs, indicated that these oligopeptides reduce the virulence of the fungi belonged to the division of Chytridiomycota, known as chytrids. This contrast may partially explain the dominance of a cyanobacterial chemotype in a determined environment, serving as a great defensive strategy to retard the parasite adaptation and to increase its diversity [16]. 

Anabaenopeptins and microviridins are most likely involved with the inhibition of the protease released by rhizoids whereas MCs are most probably related to the inactivation of their phosphatases [16]. Both groups of enzymes occupy a significant position in cell metabolism, participating in regulatory processes and signaling [132]. Strategies utilized by chytrids during infection are still an open question, but it is known that they can infect akinetes, vegetative cells, and/or heterocyst. Resource available inside the host can be one of the reasons for variations encountered among the infectivity methods. Akinetes offer higher energy and organic material content than vegetative cells. Oligopeptides distribution among the vegetative cells and those that are differentiated, such as akinetes and heterocysts should also be investigated since these secondary metabolites can sometimes be restricted to a cellular subgroup [133].

Cyanopeptides could also act as carbon and nitrogen source for various heterotrophic bacteria. These microorganisms are capable of degrading an enormous quantity of molecules with variable structures [134,135]. Besides the oligopeptides, the phycosphere is rich in carbohydrates, proteins, and lipids that originated from the exudate of cyanobacteria or its cell lysis. The mineralization of these organic compounds leads to CO_2_ production, which may contribute to the growth of cyanobacteria [136]. Briand and colleagues [137] observed that the supplementation of an axenic culture of *M. aeruginosa* PCC7806 with bacteria associated with the mucilage of *M. aeruginosa* colonies collected during a bloom eliminated all oligopeptide encountered in extracellular fraction, including MC, cyanopeptolin, and cyanobactin. In a previous investigation, Kato and co-workers [138] identified in the cell extract of the bacterium *Sphingomonas* sp. B-9 hydrolytic activity for AP A, microcyclamide, nostophycin, aeruginopeptin 95-A, and microviridin I. Anabaenopeptin A degradation was gradual and subproducts were not observed. 

APs and planktopeptin BL1125, both isolated from the bloom-forming *Planktothrix rubescens*, were associated with the collapse of cyanobacterial populations during the bloom termination. These oligopeptides act as triggers, inducing cellular lysis by virus-like particles, most likely cyanophages. Such propriety can explain in part the dominance and the high invasive potential of this species [139]. Sedmak and colleagues [67] attested it when verified that the cell growth of a non-xenic culture of *M. aeruginosa* MA2-NIB was inhibited when treated with these oligopeptides whereas the axenic were not affected. Such activity was not attributed to the known property of these peptides since the serine protease inhibitor 4-(2-aminoethyl) benzenesulfonyl fluoride hydrochloride failed in inducing any type of effect in the growth of these strains. Enrichment of the medium with the bacteria isolated from the non-axenic culture also did not produce any type of alteration. In contrast, the addition of the planktopeptin in an axenic culture of *Microcystis* previously supplemented with particulate materials obtained from cell lysate of the non-axenic culture provokes cell disintegration.

According to the hypothesis raised by these authors [67], the release of the cyclic peptides, mediated by cell lysis, signalizes the presence of a determined host and consequently activates the lytic cycle. A small concentration of these oligopeptides in the environment causes limited lysis, confined solely to a specific region. Cyanobacterial blooms offer the ideal condition for collapse since their cells are exposed to an elevated quantity of infection agents and cyclic peptides [99]. Cell-lysis provoked by cyanophages can promote the release of oligopeptide to the extracellular matrix, feeding positively the cycle [140]. In a subsequent study, algaecide property was reported for Anabaenopeptin KVJ811, which was capable of jeopardizing the growth of the strain *Nostoc* sp. KVJ11, pointing out the importance of these oligopeptides in populational control [21]. 

Given the above importance of these agents in the environment, the ecological functions of APs in the aquatic environment are much more extensive than we have already known. They are remarkably diverse in structural and functional terms. Novel Anabaenopeptins have been constantly isolated and identified. From an ecological and evolutionary perspective, these cyclopeptides allow communication with different organisms and are decisive elements in natural selection.

## 6. Applications of Anabaenopeptins 

Cyanopeptides such as APs have a well-demonstrated capacity of protease inhibition [141]. Protein Phosphatase 1 (PP1), Protein Phosphatase 2A, Carboxypeptidase-A (CPA), Human Serine Protease, Leucine Aminopeptidase, Trypsin, and Thrombin have already been tested against several cyanobacterial extracts and confirmed the catalysis blockage [11].

Among the enzymes listed above, APs were more effective inhibitors against CPA, PP1, and elastase. In a cyanobacteria bloom, it was isolated eight different Anabaenopeptins which showed activity towards CPA and protein phosphatase 1 [34,142]. PP1 inhibition may be influencing the HIV-1 transcription, cancer, or cardiac hypertrophy, for example [143,144]. Some APs half-maximal inhibitory concentration (IC_50_) values are presented in Table 3.

Serine/threonine protein phosphatases inhibition was also reported [22,25]. Nevertheless, several other cyanopeptides presented more effective IC_50_ levels against elastase, such as some variants of lyngbyastatins, symplostatins, microvirins, and others. Concerning PP1, MCs remain the best inhibitor among all cyanopeptides [11]. IC_50_ reported values to MCs and nodularins are from 1.1 to 1.9 nM as PP1 inhibitors [147]. In this case, APs remain promising candidates in Carboxypeptidase inhibition. 

Cyanopeptides blooms events may present the production of different classes of cyanopeptides like MCs, APs, and cyanopeptolins. A few studies quantified cyanopeptides beyond Microcystins, even so, in 10 eutrophic lakes in the United States and Europe the cyanopeptides concentration including these 3 types of cyanopeptides were from <4 µg/L to >40 µg/L [11]. In wet weight, 2.1 mg of AP and 7.4 mg of Microcystin-LR were obtained from 1.7 kg of biomass in a water bloom of lake Teganuma (Japan) [41].

In a study conducted by Spoof and coworkers [34], the production range of the APs measured in extracts from cyanobacteria sampled by plankton net was from 1.7 to 181.9 µg/mL in 22 isolated Anabaenopeptins. Bioactivity assays identified IC_50_ values from 16 to 435 ng/mL (Nodulapeptin 933 and Anabaenopeptin 813, respectively) against PP1 and from below 3 to 45 µg/mL against CPA (Anabaenopeptins A, D and Nodulapeptin 883C and 917: <3 µg/mL; Nodulapeptin 867: 45 µg/mL). The inhibition of elastase, trypsin, or thrombin does not occur independently of the exocyclic residue (Phe, Ile, and Tyr). The residues adjacent to the ureido bond have a major influence on CPA inhibition. Therefore, APs with Ile and Tyr in the exocyclic position presented the best IC_50_ values against this enzyme. Thus, hydrophobic aromatic or linear sidechain next to the ureido moiety presents more favorable interactions with CPA while positive amino acids such as Arg are unfavorable. It explains why Anabaenopeptin B presents IC_50_: >20 µg/mL and Anabaenopeptin 679 (different only in this position) had an improved inhibitory activity IC_50_: 4.6 µg/mL [53].

Anabaenopeptins B and F presented activity against human leukocyte elastase (HLE) and porcine pancreatic elastase (PE). Ki values of HLE inhibition were in the 0.1–1 µM range in a linear competitive model [148]. In another study, APs A and B were capable of relaxing rat aortic preparations in a concentration-dependent form using 10–400 µg/mL [20].

**Figure 13:**
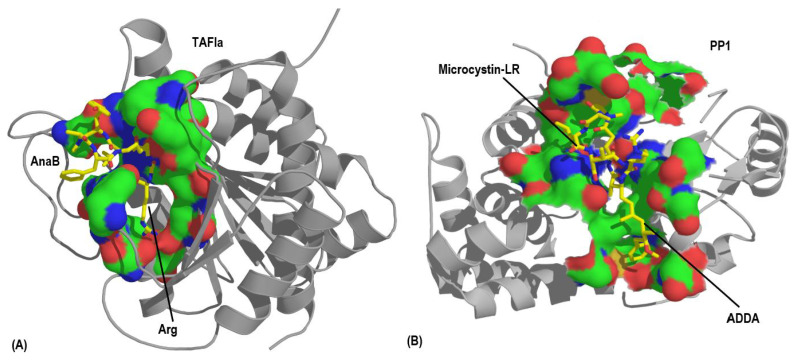
Interaction between (**A**) Anabaenopeptin B (AnaB) with Thrombin Activatable Fibrinolysis Inhibitor (TAFIa) and (**B**) the complex between Microcystin-LR and Protein Phosphatase 1 (PP1).

Some studies have been explored APs bioactive properties in a pharmaceutical/biotechnological way. Despite APs ability to inhibit diverse proteases, other cyanopeptides present the best IC_50_ values than them in most cases. However, one application shows more promising results using APs: the inhibition of the Thrombin Activatable Fibrinolysis Inhibitor (TAFIa), which is a proteolytic enzyme that cleaves Arg and Lys residues on fibrin and may be a novel antithrombotic mechanism [149]. Anabaenopeptins B, C, and F, isolated from *Planktothrix rubescens*, presented high promising results inhibiting TAFIa selectively over other coagulation enzymes as Carboxypeptidases A, B and N, FXa, FVIIa, FIIa, and FXIa [12,145]. In this sense, Anabaenopeptin B showed the best values of IC_50_ (1.5 nM, in different studies, similar to PP1 inhibition by microcystins) on a screening performed with 20 APs isolated from *Nostoc* and *Planktothrix* strains. It was elucidated that Lys and Arg residues in the R1 position (considering Anabaenopeptin B as reference: Arg-Lys-Val-Hty-MeAla-Phe) are associated with high activity (IC_50_ values of 2.1 and 1.5 nM, respectively) since those structures presenting Tyr residue in this position showed a significant decline of activity by two orders of magnitude (IC_50_ of 400 nM). In the R3 position, it was observed that the substitution of Ile by Val does not affect activity. R5 position also presents a loss of potency when the residue Ala is replaced by Ser. It is also observed a high tolerance towards substitutions in the pentacyclic region [12]. The Val to Ile difference among APs B and F does not implicate in gain or loss of activity against TAFIa. The mechanism involved in TAFIa inhibition depends on the linear part of APs mimics the carboxy−terminus of fibrin which is able of penetrating the active site pocket. Hence, the circular fraction of APs blocks the channel’s entrance, preventing the interaction with other molecules [12]. To compare the interactions of the Anabaenopeptin B-TAFIa complex with Microcystin LR-PP1 complex, TAFIa structure was obtained from Protein Data Bank (PDB), it was resolved by X-ray diffraction presenting 2,5 Å resolution (PDB code: 3LMS) (Figure 13). Also, 1.84 Å resolution PP1 in complex with Microcystin-LR was used to represent the binding mechanism (PDB code: 6OBQ). Microcystin MeAsp residue blocks the access to the PP1 active site, the long hydrophobic tail composed of Adda residues plays an important role in this inhibition due to its interaction with the hydrophobic groove region, adjacent to the catalytic site [45]. Yet, different from the linear part of Anabaenopeptin B which accesses a protein channel, Adda residue in Microcystin-LR makes contact with superficial residues of PP1 (Figure 13).

Besides some cyanopeptides presented anticancer activity, APs have been presented poor results in cytotoxic tests [150]. Anabaenopeptin B had been tested about its anticancer potential and did not demonstrate cytotoxic effects against N2a, MCF−7, and GH4 cells even at the 500 µg/mL concentration [151]. Despite anticancer activity was detected in *Aliinostoc* sp. CENA543 extract containing AP, it was not possible to attribute this effect exclusively to this class of oligopeptides because there were other cyanopeptides in the extract, and the exact AP was not identified [152]. No cytotoxic activity was presented by Nodulapeptins 883C, 869, 867, 865, and Anabaenopeptin 813 as well [34].

## 7. Final Considerations

Anabaenopeptins are structurally diverse molecules widely distributed in distinct ecosystems. Some structural features of these oligopeptides are shared with other cyanotoxins, such as the presence of modified residues, exocyclic amino acids, circular structure, and amino acids in D-configuration. However, among the cyanopeptides, the ureido linkage is exclusively found in APs. Despite their elevated occurrence and structural diversity, the majority of this group of peptides has been isolated from filamentous cyanobacteria, being commonly associated with specific genera. In freshwater environments, APs B and F are the most recurrent whereas the marine strains normally display a higher number of exclusive APs. Terrestrial cyanobacteria possess in common few APs with those from the aquatic environment.

The production of these toxins is influenced by environmental factors, which include nutrient concentration, temperature, light intensity, and association with other organisms. NRPS apparatus mediates the APs biosynthesis, following the collinearity rule of the modules. The low specificity of adenylation domains toward their substrate and the presence of additional modules are responsible for the production of various variants by a single strain. Homoamino acids present in the APs structure are supplied by HphABCD biosynthetic pathway. Some modifications are catalyzed by specific domains encountered in the NRPS modules. However, the mechanism of ureido bond formation is still unknown. 

APs have been increasingly detected in reservoirs, lakes, and oceans in very elevated concentrations. Their toxicity for human beings has not yet been determined. However, assays employing animal models as well as other organisms have demonstrated their deleterious actions. In face of this fact, there is a dire need to further investigate the real impact of these oligopeptides on human health. The inhibitory activity of these molecules against proteases, phosphatases, and carboxylases makes them very promising for biotechnological use, but their mechanisms of action need to be investigated in detail to be properly applied.

However, Anabaenopeptins still require further studies to comprehend their behaviors in nature. Among AP producers, it must be evaluated the evolutionary relationship between terrestrial and aquatic strains as they do not share a high number of AP variants, similar to freshwater and marine. Besides, specific residues are more predominantly found in some environments, requiring additional analysis to comprehend the relationship between their frequency and habitats. Moreover, just a few variants have been analyzed regarding their inhibitory properties, then demanding more tests to discern the role of specific amino acids during the interaction with their targets. Another issue that must be investigated deeply is the APs amount produced by cyanobacteria. Their low yield can be a limited factor for industrial purposes. Such bottlenecks can be minimized through the use of heterologous expression, which has been well established for other cyanopeptides. 

## Figures and Tables

**Table 1:** Amino acid composition of anabaenopeptin-like peptides obtained from sponges. Amino acids are considered in L-configuration unless otherwise defined. Ala: Alanine; Arg: Arginine; Ile: Isoleucine; Leu: Leucine; Lys: Lysine; MeLeu: N-methyl-Leucine; Phe: Phenylalanine; Trp: Tryptophan; Allo-Ile: Allo-Isoleucine; 2’BrTrp: 2-bromo-5-hydroxytryptophan; NMe-6’ClTrp: 6-chloro-N-methyltryptophan; 5’OHTrp: 5’-hydroxytryptophan; 6’BrTrp: 6’-bromotryptophan; 5’OH-6’Cl Trp: 6’-chloro-5’-hydroxytryptophan; 6’ClTrp: 6’-chloro-tryptophan; NMe-5OHTrp: N-methyl-5’-hydroxytryptophan; NMe-5’BrTrp: 5’-Bromo-N-methyltryptophan.

Nomenclature		Position						Reference
		1	2	3	4	5	6	
Konbamide	-	Leu	L-Lys	Ala	Leu	MeLeu	2’BrTrp	[31]
Keramide	A	Phe	L-Lys	Leu	Leu	5’OH-6’ClTrp	Phe	[32]
	L	Phe	L-Lys	Leu	Leu	NMe-6′ClTrp	Phe	[30]
Paltolide	A	Arg	D-Lys	Ala	Leu	Leu	Trp	[28]
	B	Arg	D-Lys	Ala	Leu	MeLeu	5’OHTrp	[28]
	C	Arg	D-Lys	Ala	Leu	MeLeu	6’BrTrp	[28]
Unnamed	1	Arg	D-Lys	Ala	Leu	MeLeu	5’OH-6’ClTrp	[28,51]
	2	Arg	D-Lys	Ala	Leu	MeLeu	6’ClTrp	[28,51]
	3	Arg	D-Lys	Ala	Leu	MeLeu	Trp	[28,51]
Mozamide	A	Allo-Ile	L-Lys	D-Val	Leu	NMe-5OHTrp	Phe	[29]
	B	Allo-Ile	L-Lys	D-Ile	Leu	NMe-5OHTrp	Phe	[29]
Psymbamide	A	Ile	D-Lys	Leu	Leu	NMe-5’BrTrp	Phe	[33]

**Table 2:** Occurrence of anabaenopeptins in different cyanobacteria genera and species.

Strains	Anabaenopeptin	Reference
***Freshwater***		
*Anabaena flos-aquae* 202 A 1	Anabaenopeptins B and D	[44]
*Anabaena flos-aquae* CYA 83/1	Anabaenopeptins B and D	[44]
*Anabaena lemmermannii* 202 A2/41	Anabaenopeptins B and C	[44]
*Aphanizomenon flos-aquae* NIES-81	Anabaenopeptins I and J	[42]
*Lyngbya* sp. (SAG 36.91)	Lyngbyaureamide A and B	[47]
*Microcystis aeruginosa* HUB 063	Anabaenopeptins B and F	[40]
*Microcystis aeruginosa* Kutz	Ferintoic acids A and B	[39]
*Microcystis aeruginosa* PCC7806	Anabaenopeptins A, B, E/F and Oscillamide Y	[60]
*Microcystis aeruginosa* TAU IL-342	Anabaenopeptin HU892	[61]
*Microcystis* sp. (MB-K)	Anabaenopeptin KT864	[52]
*Microcystis* sp. TAU IL-306	Anabaenopeptin F and Oscillamide Y	[61]
*Microcystis* sp. TAU IL-362	Anabaenopeptins MM823, MM850, MM913 and B	[61]
*Microcystis* spp.	Anabaenopeptin KB905, KB899, G, H, 908A, 915, HU892, MM913	[62]
*Nodularia spumigena* Node 2	Nodulapeptins B, C, 855B, 871, 879, 897 and 915A	[14,49]
*Nodularia spumigena* Nodg 3	Nodulapeptins B, C, 855B, 871, 879, 897 and 915A	[14]
*Nodularia spumigena* Nodh 2	Nodulapeptins B, C, 855B, 871, 879, 897 and 915A	[14]
*Nodularia spumigena* NSBL-05	Anabaenopeptin 807	[14]
*Nodularia spumigena* NSBL-06	Anabaenopeptin 807	[14]
*Nodularia spumigena* NSBR-01	Anabaenopeptin 807	[14]
*Nodularia spumigena* NSGL-01	Anabaenopeptin 807	[14]
*Nodularia spumigena* NSKR-07	Anabaenopeptin 807	[14]
*Nodularia spumigena* NSLA-01	Anabaenopeptin 807	[14]
*Nodularia spumigena* NSOR-02	Anabaenopeptin 807	[14]
*Nodularia spumigena* NSPH-02	Anabaenopeptin 807	[14]
*Oscillatoria agardhii* CYA 128	Anabaenopeptins A and C	[44]
*Oscillatoria agardhii* NIES-204	Anabaenopeptins B, E and F	[38]
*Oscillatoria agardhii* NIES-595	Anabaenopeptin G and H	[26]
*Planktothrix agardhii* CCAP 1459/11A	Anabaenopeptin F and Oscillamide B	[25]
*Planktothrix agardhii* CYA126/8	Anabaenopeptin 908A and 915	[63]
*Planktothrix agardhii* HUB 011	Anabaenopeptin G	[40]
*Planktothrix agardhii* NIVA CYA 15	Anabaenopeptins A and B	[64]
*Planktothrix agardhii* NIVA CYA 34	Anabaenopeptins A, B, F and Oscillamide Y	[64]
*Planktothrix mougeotii* NIVA CYA 405	Anabaenopeptins A, B, F and Oscillamide Y	[64]
*Planktothrix mougeotii* NIVA CYA 56/3	Anabaenopeptins C, 822 *, B, and F	[64]
*Planktothrix prolifica* NIVA CYA 406	Anabaenopeptins A, B, F and Oscillamide Y	[64]
*Planktothrix prolifica* NIVA CYA 540	Anabaenopeptins A, B, F and Oscillamide Y	[64]
*Planktothrix prolifica* NIVA CYA 98	Anabaenopeptins A, B, F and Oscillamide Y	[18,64]
*Planktothrix rubescens*	Anabaenopeptins A, B, F and Oscillamide Y	[65]
*Planktothrix rubescens*	Anabaenopeptins A, B, C, F and Oscillamide Y	[66]
*Planktothrix rubescens*	Anabaenopeptins B and F	[67]
*Planktothrix rubescens*	Anabaenopeptin A, B, and F	[68]
*Planktothrix rubescens* BGSD-500	Anabaenopeptins B and F	[69]
*Planktothrix rubescens* NIES-610	Anabaenopeptin F	[25]
*Planktothrix rubescens* NIVA CYA 407	Anabaenopeptins C, 822 *, B, and F	[64]
*Woronichinia naegeliana*	Anabaenopeptin 899	[70]
***Marine***		
*Anabaena* sp. TAU NZ-3-1	Anabaenopeptins NZ841, NZ825 and NZ857	[71]
*Coelosphaeriaceae* cyanobacterium 06S067	Anabaenopeptins A, B, F, 802 *, 827 *, 809 * and Oscillamide Y	[72]
*Nodularia spumigena* AV1	Nodulapeptins A, B, C, 871, 821, 839, 849, 855A, 863, 865, 867, 879, 881A, 881B, 883A, 897, 899A, 915A, 931	[14,48,49]
*Nodularia spumigena* B15a	Anabaenopeptins 841 and D	[14]
*Nodularia spumigena* BY1	Anabaenopeptin B and Nodulapeptins B, C, 821, 839, 855A, 855B, 871, 879, 881A, 881B, 883A, 897, 899A, 915A, 931	[14,48,49]
*Nodularia spumigena* CCNP 1401	Anabaenopeptins 841A and D	[14,49]
*Nodularia spumigena* CCNP 1423	Nodulapeptins 883B, 899B, 901, 915B, 917, 933	[14,49]
*Nodularia spumigena* CCNP 1424	Nodulapeptins 883B, 899B, 901, 915B, 917, 933	[14,49]
*Nodularia spumigena* CCNP 1425	Nodulapeptins 883B, 899B, 901, 915B, 917, 933	[14,49]
*Nodularia spumigena* CCNP 1402	and Nodulapeptins A, B, C, 821, 839, 855A, 855B, 871, 879, 881A, 881B, 883A, 897, 899A, 915A, 931	[14,49]
*Nodularia spumigena* CCNP 1403	Anabaenopeptins 841A and D	[14,49]
*Nodularia spumigena* CCNP 1426	Anabaenopeptins D and 841A	[49]
*Nodularia spumigena* CCNP 1427	Nodulapeptins B, C, 821, 855A, 855B, 871, 879, 881A, 881B, 883A, 897, 899A, 915A and 931	[49]
*Nodularia spumigena* CCNP 1428	Nodulapeptins 883B, 899B, 901, 915B, 917 and 933	[49]
*Nodularia spumigena* CCNP 1430	Anabaenopeptins D and 841A	[49]
*Nodularia spumigena* CCNP 1431	Nodulapeptins 883B, 885, 899B, 901, 915B, 917 and 933	[49]
*Nodularia spumigena* CCNP 1436	Nodulapeptins B, C, 839, 855A, 855B, 871, 879, 881A, 881B, 883A, 897, 899A, 915A, 921 and 931	[49]
*Nodularia spumigena* CCNP 1440	Nodulapeptins 883B, 885, 899B, 901, 915B, 917 and 933	[49]
*Nodularia spumigena* CCY 9414	Nodulapeptins A, B, C, 839, 855A, 855B, 871, 879, 881A, 881B, 883A, 897, 899A, 915A, 931	[14,49,73]
*Nodularia spumigena* KAC 11	Anabaenopeptins J and 807	[49]
*Nodularia spumigena* KAC 13	Anabaenopeptins D and 841A	[49]
*Nodularia spumigena* KAC 64	Nodulapeptins 883B, 885, 899B, 901, 915B, 917 and 933	[49]
*Nodularia spumigena* KAC 66	Nodulapeptins 883B, 885, 857, 899B, 901, 915B, 917 and 933	[14,49]
*Nodularia spumigena* KAC 68	Nodulapeptins 883B, 885, 857, 899B, 901, 917 and 933	[49]
*Nodularia spumigena* KAC 7	Nodulapeptins B, C, 921, 839, 855A, 855B, 871, 879, 881A, 881B, 883A, 897, 899A, 915A and 931	[49]
*Nodularia spumigena* KAC 70	Nodulapeptins 807, 823, 851, 865, 867 and 883C	[49]
*Nodularia spumigena* KAC 71	Nodulapeptins A, B, C, 921, 823, 839, 855A, 855B, 871, 879, 881A, 881B, 883A, 897, 899A, 915A and 931	[49]
*Nodularia spumigena* KAC 87	Nodulapeptins 807, 823, 849, 851, 865, 867 and 883C	[49]
*Nodularia spumigena* UHCC0039	Nodulapeptins A, B, C, 839, 849, 855A, 863, 865, 867, 871, 879, 881A, 881B, 897, 899A, 915A and 933	[73]
***Terrestrial***		
*Anabaena circinalis* 90	Anabaenopeptins A, B, and C	[44]
*Anabaena flos-aquae* NRC 525-17	Anabaenopeptins A and B	[20]
*Brasilonema* sp. 360	Anabaenopeptin 802A	[24]
*Brasilonema* sp. 382	Anabaenopeptin 802A	[24]
*Brasilonema* sp. CT11	Anabaenopeptins 788, 802A, 802B and 816	[74]
*Desmonostoc* sp. 386	Anabaenopeptins 848, 849, 862, 863, 877A, 877B, 891 and 905	[24]
*Nostoc* sp. 352	Anabaenopeptins 841B, 855, 857 and 871	[24]
*Nostoc* sp. 358	Anabaenopeptins 882 and 896	[24]
*Nostoc* sp. ASN_M	Anabaenopeptins 808 *, 828, 842 *, 844 * and 858 *,	[75]
*Nostoc* sp. ATCC 53789	Anabaenopeptin SA9, SA10, SA11 and SA12	[12]
*Nostoc* sp. KVJ2	Anabaenopeptins KVJ827, KVJ841, and KVJ811	[21]
*Schizothrix sp.* IL-208-2-2	Schizopeptin 791	[46]

* Anabaenopeptin variants with non-elucidated sequence.

**Table 3:** Detected IC_50_ values of some Anabaenopeptins in nM. (TAFIa: Thrombin Activatable Fibrinolysis Inhibitor; PP1: Protein Phosphatase 1).

Anabaenopeptin	TAFIa (nM)	Carboxipeptidase A (nM)	PP1 (nM)	References
Anabaenopeptin 679	-	6740	-	[53]
Anabaenopeptin 908A	1.8	>11,000	-	[12,53]
Anabaenopeptin 915	530	130	-	[12,53]
Anabaenopeptin A	440	-	104,300	[12,34]
Anabaenopeptin B	1.5	>60,000; 3900	119,500	[12,145,146]
Anabaenopeptin C	1.9	>100,000	-	[12,145]
Anabaenopeptin E	-	>60,000	-	[53]
Anabaenopeptin F	1.5	>60,000; 1100	44770	[12,25,53]
Anabaenopeptin G	-	2–8	-	[53]
Anabaenopeptin H	-	3700–10,200	-	[53]
Anabaenopeptin I	-	7	-	[53]
Anabaenopeptin J	-	10	-	[53]
Anabaenopeptin SA1	2.2	-	-	[12]
Anabaenopeptin SA10	7000	-	-	[12]
Anabaenopeptin SA11	15000	-	-	[12]
Anabaenopeptin SA12	4300	-	-	[12]
Anabaenopeptin SA13	2500	-	-	[12]
Anabaenopeptin SA2	16	-	-	[12]
Anabaenopeptin SA3	2.1	-	-	[12]
Anabaenopeptin SA4	3.4	-	-	[12]
Anabaenopeptin SA5	790	-	-	[12]
Anabaenopeptin SA6	51000	-	-	[12]
Anabaenopeptin SA7	13000	-	-	[12]
Anabaenopeptin SA8	4800	-	-	[12]
Anabaenopeptin SA9	31000	-	-	[12]
Anabaenopeptin T	-	3–2300	-	[53]
Oscillamide Y	400	-	72.26	[12,34]

## Data Availability

Not applied.

## Supplementary Materials

The following are available online at https://www.mdpi.com/article/10.3390/toxins13080522/s1, Table S1: Sequence composition of Anabaenopeptins.

## References

[1] do Amaral S.C., Santos A.V., da Cruz Schneider M.P., da Silva J.K.R., Xavier L.P. (2020). Determination of Volatile Organic Compounds and Antibacterial Activity of the Amazonian Cyanobacterium Synechococcus sp. Strain GFB01. Molecules.

[2] Gradíssimo D.G., Xavier L.P., Santos A.V. (2020). Cyanobacterial Polyhydroxyalkanoates: A Sustainable Alternative in Circular Economy. Molecules.

[3] de Oliveira D.T., da Costa A.A.F., Costa F.F., da Rocha Filho G.N., do Nascimento L.A.S. (2020). Advances in the Biotechnological Potential of Brazilian Marine Microalgae and Cyanobacteria. Molecules.

[4] Huisman J., Codd G.A., Paerl H.W., Ibelings B.W., Verspagen J.M.H., Visser P.M. (2018). Cyanobacterial blooms. Nat. Rev. Microbiol..

[5] Bittner M., Štern A., Smutná M., Hilscherová K., Žegura B. (2021). Cytotoxic and Genotoxic Effects of Cyanobacterial and Algal Extracts—Microcystin and Retinoic Acid Content. Toxins.

[6] Chen G., Wang L., Li W., Zhang Q., Hu T. (2020). Nodularin induced oxidative stress contributes to developmental toxicity in zebrafish embryos. Ecotoxicol. Environ. Saf..

[7] Zhu H., Sonoyama T., Yamada M., Gao W., Tatsuno R., Takatani T., Arakawa O. (2020). Co-Occurrence of Tetrodotoxin and Saxitoxins and Their Intra-Body Distribution in the Pufferfish Canthigaster valentini. Toxins.

[8] Moosová Z., Šindlerová L., Ambrůzová B., Ambrožová G., Vašíček O., Velki M., Babica P., Kubala L. (2019). Lipopolysaccharides from Microcystis Cyanobacteria-Dominated Water Bloom and from Laboratory Cultures Trigger Human Immune Innate Response. Toxins.

[9] Massey I.Y., Yang F. (2020). A Mini Review on Microcystins and Bacterial Degradation. Toxins.

[10] Jones M.R., Pinto E., Torres M.A., Dörr F., Mazur-Marzec H., Szubert K., Tartaglione L., Dell’Aversano C., Miles C.O., Beach D.G. (2021). CyanoMetDB, a comprehensive public database of secondary metabolites from cyanobacteria. Water Res..

[11] Janssen E.M.L. (2019). Cyanobacterial peptides beyond microcystins—A review on co-occurrence, toxicity, and challenges for risk assessment. Water Res..

[12] Schreuder H., Liesum A., Lönze P., Stump H., Hoffmann H., Schiell M., Kurz M., Toti L., Bauer A., Kallus C. (2016). Isolation, Co-Crystallization and Structure-Based Characterization of Anabaenopeptins as Highly Potent Inhibitors of Activated Thrombin Activatable Fibrinolysis Inhibitor (TAFIa). Sci. Rep..

[13] do Amaral S.C., Monteiro P.R., da Neto J.S.P., Serra G.M., Gonçalves E.C., Xavier L.P., Santos A.V. (2021). Current Knowledge on Microviridin from Cyanobacteria. Mar. Drugs.

[14] Mazur-Marzec H., Kaczkowska M., Blaszczyk A., Akcaalan R., Spoof L., Meriluoto J. (2013). Diversity of Peptides Produced by Nodularia spumigena from Various Geographical Regions. Mar. Drugs.

[15] Gkelis S., Lanaras T., Sivonen K. (2015). Cyanobacterial Toxic and Bioactive Peptides in Freshwater Bodies of Greece: Concentrations, Occurrence Patterns, and Implications for Human Health. Mar. Drugs.

[16] Rohrlack T., Christiansen G., Kurmayer R. (2013). Putative Antiparasite Defensive System Involving Ribosomal and Nonribosomal Oligopeptides in Cyanobacteria of the Genus Planktothrix. Appl. Environ. Microbiol..

[17] Martins J., Vasconcelos V. (2015). Cyanobactins from Cyanobacteria: Current Genetic and Chemical State of Knowledge. Mar. Drugs.

[18] Rounge T.B., Rohrlack T., Nederbragt A.J., Kristensen T., Jakobsen K.S. (2009). A genome-wide analysis of nonribosomal peptide synthetase gene clusters and their peptides in a Planktothrix rubescens strain. BMC Genom..

[19] Welker M., Von Döhren H. (2006). Cyanobacterial peptides—Nature’s own combinatorial biosynthesis. FEMS Microbiol. Rev..

[20] Harada K.I., Fujii K., Shimada T., Suzuki M., Sano H., Adachi K., Carmichael W.W. (1995). Two cyclic peptides, anabaenopeptins, a third group of bioactive compounds from the cyanobacteriumAnabaena flos-aquae NRC 525-17. Tetrahedron Lett..

[21] Guljamow A., Kreische M., Ishida K., Liaimer A., Altermark B., Bähr L., Hertweck C., Ehwald R., Dittmann E. (2017). High-Density Cultivation of Terrestrial Nostoc Strains Leads to Reprogramming of Secondary Metabolome. Appl. Environ. Microbiol..

[22] Zafrir-Ilan E., Carmeli S. (2010). Eight novel serine proteases inhibitors from a water bloom of the cyanobacterium *Microcystis* sp.. Tetrahedron.

[23] Matthew S., Ross C., Paul V.J., Luesch H. (2008). Pompanopeptins A and B, new cyclic peptides from the marine cyanobacterium Lyngbya confervoides. Tetrahedron.

[24] Sanz M., Andreote A.P.D., Fiore M.F., Dörr F.A., Pinto E. (2015). Structural characterization of new peptide variants produced by cyanobacteria from the Brazilian Atlantic Coastal Forest using liquid chromatography coupled to quadrupole time-of-flight tandem mass spectrometry. Mar. Drugs.

[25] Sano T., Usui T., Ueda K., Osada H., Kaya K. (2001). Isolation of new protein phosphatase inhibitors from two cyanobacteria species, *Planktothrix* spp.. J. Nat. Prod..

[26] Itou Y., Suzuki S., Ishida K., Murakami M. (1999). Anabaenopeptins G and H, potent carboxypeptidase A inhibitors from the cyanobacterium *Oscillatoria agardhii* (NIES-595). Bioorganic Med. Chem. Lett..

[27] Tonk L., Welker M., Huisman J., Visser P.M. (2009). Production of cyanopeptolins, anabaenopeptins, and microcystins by the harmful cyanobacteria Anabaena 90 and Microcystis PCC 7806. Harmful Algae.

[28] Plaza A., Keffer J.L., Lloyd J.R., Colin P.L., Bewley C.A. (2010). Paltolides A−C, Anabaenopeptin-Type Peptides from the Palau Sponge *Theonella swinhoei*. J. Nat. Prod..

[29] Schmidt E.W., Harper M.K., Faulkner D.J. (1997). Mozamides A and B, cyclic peptides from a theonellid sponge from Mozambique. J. Nat. Prod..

[30] Uemoto H., Yahiro Y., Shigemori H., Tsuda M., Takao T., Shimonishi Y., Kobayashi J. (1998). Keramamides K and L, new cyclic peptides containing unusual tryptophan residue from *Theonella sponge*. Tetrahedron.

[31] Kobayashi J., Sato M., Murayama T., Ishibashi M., Wälchi M.R., Kanai M., Shoji J., Ohizumi Y. (1991). Konbamide, a novel peptide with calmodulin antagonistic activity from the Okinawan marine sponge Theonella sp.. J. Chem. Soc. Chem. Commun..

[32] Kobayashi J., Sato M., Ishibashi M., Shigemori H., Nakamura T., Ohizumi Y. (1991). Keramamide A, a novel peptide from the Okinawan marine sponge *Theonella* sp.. J. Chem. Soc. Perkin Trans..

[33] Robinson S.J., Tenney K., Yee D.F., Martinez L., Media J.E., Valeriote F.A., van Soest R.W.M., Crews P. (2007). Probing the Bioactive Constituents from Chemotypes of the Sponge *Psammocinia* aff. bulbosa. J. Nat. Prod..

[34] Spoof L., Błaszczyk A., Meriluoto J., Cegłowska M., Mazur-Marzec H. (2015). Structures and Activity of New Anabaenopeptins Produced by Baltic Sea Cyanobacteria. Mar. Drugs.

[35] Greunke C., Duell E.R., D’Agostino P.M., Glöckle A., Lamm K., Gulder T.A.M. (2018). Direct Pathway Cloning (DiPaC) to unlock natural product biosynthetic potential. Metab. Eng..

[36] Sano T., Kaya K. (1995). Oscillamide Y, a chymotrypsin inhibitor from toxic *Oscillatoria agardhii*. Tetrahedron Lett..

[37] Murakami M., Shin H.J., Matsuda H., Ishida K., Yamaguchi K. (1997). A cyclic peptide, anabaenopeptin B, from the cyanobacterium *Oscillatoria agardhii*. Phytochemistry.

[38] Shin H.J., Matsuda H., Murakami M., Yamaguchi K. (1997). Anabaenopeptins E and F, two new cyclic peptides from the cyanobacterium *Oscillatoria agardhii* (NIES-204). J. Nat. Prod..

[39] Williams D.E., Craig M., Holmes C.F.B., Andersen R.J. (1996). Ferintoic acids A and B, new cyclic hexapeptides from the freshwater cyanobacterium *Microcystis aeruginosa*. J. Nat. Prod..

[40] Erhard M., Von Döhren H., Jungblut P.R. (1999). Rapid identification of the new anabaenopeptin G from *Planktothrix agardhii* HUB 011 using matrix-assisted laser desorption/ionization time-of-flight mass spectrometry. Rapid Commun. Mass Spectrom..

[41] Kodani S., Suzuki S., Ishida K., Murakami M. (1999). Five new cyanobacterial peptides from water bloom materials of lake Teganuma (Japan). FEMS Microbiol. Lett..

[42] Murakami M., Suzuki S., Itou Y., Kodani S., Ishida K. (2000). New Anabaenopeptins, Potent Carboxypeptidase-A Inhibitors from the Cyanobacterium Aphanizomenon flos-aquae. J. Nat. Prod..

[43] Mi Y., Zhang J., He S., Yan X. (2017). New Peptides Isolated from Marine Cyanobacteria, an Overview over the Past Decade. Mar. Drugs.

[44] Fujii K., Harada K., Suzuki M., Kondo F., Ikai Y., Oka H., Sivonen K. (1995). Novel cyclic peptides together with microcystins produced by toxic cyanobacteria, *Anabaena* sp.. Symp. Chem. Nat. Prod. Symp. Pap..

[45] Campos A., Vasconcelos V. (2010). Molecular mechanisms of microcystin toxicity in animal cells. Int. J. Mol. Sci..

[46] Reshef V., Carmeli S. (2002). Schizopeptin 791, a new anabeanopeptin-like cyclic peptide from the cyanobacterium *Schizothrix* sp.. J. Nat. Prod..

[47] Zi J., Lantvit D.D., Swanson S.M., Orjala J. (2012). Lyngbyaureidamides A and B, two anabaenopeptins from the cultured freshwater cyanobacterium *Lyngbya* sp. (SAG 36.91). Phytochemistry.

[48] Fujii K., Sivonen K., Adachi K., Noguchi K., Sano H., Hirayama K., Suzuki M., Harada K. (1997). Comparative study of toxic and non-toxic cyanobacterial products: Novel peptides from toxic *Nodularia spumigena* AV1. Tetrahedron Lett..

[49] Mazur-Marzec H., Bertos-Fortis M., Toruńska-Sitarz A., Fidor A., Legrand C. (2016). Chemical and genetic diversity of nodularia spumigena from the baltic sea. Mar. Drugs.

[50] Müller D., Krick A., Kehraus S., Mehner C., Hart M., Küpper F.C., Saxena K., Prinz H., Schwalbe H., Janning P. (2006). Brunsvicamides A-C: Sponge-related cyanobacterial peptides with Mycobacterium tuberculosis protein tyrosine phosphatase inhibitory activity. J. Med. Chem..

[51] Bjoerquist P., Buchanan M., Campitelli M., Carroll A., Hyde E., Neve J., Polla M., Quinn R. (2005). Use of cyclic anabaenopeptin-type peptides for the treatment of a condition wherein inhibition of carboxypeptidase U is beneficial, novel anabaenopeptin derivatives and intermediates thereof. U.S. Patent.

[52] Beresovsky D., Hadas O., Livne A., Sukenik A., Kaplan A., Carmeli S. (2006). Toxins and Biologically Active Secondary Metabolites of Microcystis sp. isolated from Lake Kinneret. Isr. J. Chem..

[53] Harms H., Kurita K.L., Pan L., Wahome P.G., He H., Kinghorn A.D., Carter G.T., Linington R.G. (2016). Discovery of anabaenopeptin 679 from freshwater algal bloom material: Insights into the structure–activity relationship of anabaenopeptin protease inhibitors. Bioorganic Med. Chem. Lett..

[54] Cheruku P., Plaza A., Lauro G., Keffer J., Lloyd J.R., Bifulco G., Bewley C.A. (2012). Discovery and Synthesis of Namalide Reveals a New Anabaenopeptin Scaffold and Peptidase Inhibitor. J. Med. Chem..

[55] Sanz M., Salinas R.K., Pinto E. (2017). Namalides B and C and Spumigins K-N from the Cultured Freshwater Cyanobacterium Sphaerospermopsis torques-reginae. J. Nat. Prod..

[56] Shishido T.K., Jokela J., Fewer D.P., Wahlsten M., Fiore M.F., Sivonen K. (2017). Simultaneous Production of Anabaenopeptins and Namalides by the Cyanobacterium *Nostoc* sp. CENA543. ACS Chem. Biol..

[57] Entfellner E., Frei M., Christiansen G., Deng L., Blom J., Kurmayer R. (2017). Evolution of Anabaenopeptin Peptide Structural Variability in the Cyanobacterium Planktothrix. Front. Microbiol..

[58] Wang H., Fewer D.P., Sivonen K. (2011). Genome Mining Demonstrates the Widespread Occurrence of Gene Clusters Encoding Bacteriocins in Cyanobacteria. PLoS ONE.

[59] Hallenbeck P.C. (2012). Microbial Technologies in Advanced Biofuels Production.

[60] Natumi R., Janssen E.M.L. (2020). Cyanopeptide Co-Production Dynamics beyond Mirocystins and Effects of Growth Stages and Nutrient Availability. Environ. Sci. Technol..

[61] Gesner-Apter S., Carmeli S. (2009). Protease Inhibitors from a Water Bloom of the Cyanobacterium Microcystis aeruginosa. J. Nat. Prod..

[62] Elkobi-Peer S., Carmeli S. (2015). New Prenylated Aeruginosin, Microphycin, Anabaenopeptin and Micropeptin Analogues from a Microcystis Bloom Material Collected in Kibbutz Kfar Blum, Israel. Mar. Drugs.

[63] Okumura H.S., Philmus B., Portmann C., Hemscheidt T.K. (2009). Homotyrosine-Containing Cyanopeptolins 880 and 960 and Anabaenopeptins 908 and 915 from Planktothrix agardhii CYA 126/8. J. Nat. Prod..

[64] Tooming-Klunderud A., Sogge H., Rounge T.B., Nederbragt A.J., Lagesen K., Glöckner G., Hayes P.K., Rohrlack T., Jakobsen K.S. (2013). From Green to Red: Horizontal Gene Transfer of the Phycoerythrin Gene Cluster between Planktothrix Strains. Appl. Environ. Microbiol..

[65] Welker M., Erhard M. (2007). Consistency between chemotyping of single filaments ofPlanktothrix rubescens (cyanobacteria) by MALDI-TOF and the peptide patterns of strains determined by HPLC-MS. J. Mass Spectrom..

[66] Rohrlack T., Edvardsen B., Skulberg R., Halstvedt C.B., Utkilen H.C., Ptacnik R., Skulberg O.M. (2008). Oligopeptide chemotypes of the toxic freshwater cyanobacterium Planktothrix can form sub-populations with dissimilar ecological traits. Limnol. Oceanogr..

[67] Sedmak B., Carmeli S., Eleršek T. (2008). “Non-Toxic” Cyclic Peptides Induce Lysis of Cyanobacteria—An Effective Cell Population Density Control Mechanism in Cyanobacterial Blooms. Microb. Ecol..

[68] Grach-Pogrebinsky O., Sedmak B., Carmeli S. (2003). Protease inhibitors from a Slovenian Lake Bled toxic waterbloom of the cyanobacterium *Planktothrix rubescens*. Tetrahedron.

[69] Vasas G., Farkas O., Borics G., Felföldi T., Sramkó G., Batta G., Bácsi I., Gonda S. (2013). Appearance of Planktothrix rubescens Bloom with [D-Asp3, Mdha7]MC–RR in Gravel Pit Pond of a Shallow Lake-Dominated Area. Toxins.

[70] Bober B., Chrapusta-Srebrny E., Bialczyk J. (2020). Novel cyanobacterial metabolites, cyanopeptolin 1081 and anabaenopeptin 899, isolated from an enrichment culture dominated by Woronichinia naegeliana (Unger) Elenkin. Eur. J. Phycol..

[71] Grach-Pogrebinsky O., Carmeli S. (2008). Three novel anabaenopeptins from the cyanobacterium Anabaena sp.. Tetrahedron.

[72] Häggqvist K., Toruńska-Sitarz A., Błaszczyk A., Mazur-Marzec H., Meriluoto J. (2016). Morphologic, Phylogenetic and Chemical Characterization of a Brackish Colonial Picocyanobacterium (Coelosphaeriaceae) with Bioactive Properties. Toxins.

[73] Popin R.V., Delbaje E., de Abreu V.A.C., Rigonato J., Dörr F.A., Pinto E., Sivonen K., Fiore M.F. (2020). Genomic and Metabolomic Analyses of Natural Products in Nodularia spumigena Isolated from a Shrimp Culture Pond. Toxins.

[74] Saha S., Esposito G., Urajová P., Mareš J., Ewe D., Caso A., Macho M., Delawská K., Kust A., Hrouzek P. (2020). Discovery of Unusual Cyanobacterial Tryptophan-Containing Anabaenopeptins by MS/MS-Based Molecular Networking. Molecules.

[75] Nowruzi B., Khavari-Nejad R.-A., Sivonen K., Kazemi B., Najafi F., Nejadsattari T. (2012). Identification and toxigenic potential of a *Nostoc* sp.. Algae.

[76] Grabowska M., Kobos J., Toruńska-Sitarz A., Mazur-Marzec H. (2014). Non-ribosomal peptides produced by Planktothrix agardhii from Siemianówka Dam Reservoir SDR (northeast Poland). Arch. Microbiol..

[77] de Carvalho L.R., Pipole F., Werner V.R., Laughinghous H.D., de Camargo A.C.M., Rangel M., Konno K., Sant’ Anna C.L. (2008). A toxic cyanobacterial bloom in an urban coastal lake, Rio Grande do Sul state, Southern Brazil. Braz. J. Microbiol..

[78] Teta R., Della Sala G., Glukhov E., Gerwick L., Gerwick W.H., Mangoni A., Costantino V. (2015). Combined LC–MS/MS and Molecular Networking Approach Reveals New Cyanotoxins from the 2014 Cyanobacterial Bloom in Green Lake, Seattle. Environ. Sci. Technol..

[79] Beversdorf L., Weirich C., Bartlett S., Miller T. (2017). Variable Cyanobacterial Toxin and Metabolite Profiles across Six Eutrophic Lakes of Differing Physiochemical Characteristics. Toxins.

[80] Bartlett S.L., Brunner S.L., Klump J.V., Houghton E.M., Miller T.R. (2018). Spatial analysis of toxic or otherwise bioactive cyanobacterial peptides in Green Bay, Lake Michigan. J. Great Lakes Res..

[81] Roy-Lachapelle A., Solliec M., Sauvé S., Gagnon C. (2019). A Data-Independent Methodology for the Structural Characterization of Microcystins and Anabaenopeptins Leading to the Identification of Four New Congeners. Toxins.

[82] Flores C., Caixach J. (2020). High Levels of Anabaenopeptins Detected in a Cyanobacteria Bloom from N.E. Spanish Sau-Susqueda-El Pasteral Reservoirs System by LC–HRMS. Toxins.

[83] Kust A., Řeháková K., Vrba J., Maicher V., Mareš J., Hrouzek P., Chiriac M.-C., Benedová Z., Tesařová B., Saurav K. (2020). Insight into Unprecedented Diversity of Cyanopeptides in Eutrophic Ponds Using an MS/MS Networking Approach. Toxins.

[84] Riba M., Kiss-Szikszai A., Gonda S., Boros G., Vitál Z., Borsodi A.K., Krett G., Borics G., Ujvárosi A.Z., Vasas G. (2019). Microcystis Chemotype Diversity in the Alimentary Tract of Bigheaded Carp. Toxins.

[85] Konkel R., Toruńska-Sitarz A., Cegłowska M., Ežerinskis Ž., Šapolaitė J., Mažeika J., Mazur-Marzec H. (2020). Blooms of Toxic Cyanobacterium Nodularia spumigena in Norwegian Fjords During Holocene Warm Periods. Toxins.

[86] Logares R., Bråte J., Bertilsson S., Clasen J.L., Shalchian-Tabrizi K., Rengefors K. (2009). Infrequent marine-freshwater transitions in the microbial world. Trends Microbiol..

[87] Boopathi T., Ki J.-S. (2014). Impact of Environmental Factors on the Regulation of Cyanotoxin Production. Toxins.

[88] Repka S., Koivula M., Harjunpä V., Rouhiainen L., Sivonen K. (2004). Effects of Phosphate and Light on Growth of and Bioactive Peptide Production by the Cyanobacterium Anabaena Strain 90 and Its Anabaenopeptilide Mutant. Appl. Environ. Microbiol..

[89] Tonk L., Visser P.M., Christiansen G., Dittmann E., Snelder E.O.F.M., Wiedner C., Mur L.R. (2005). The Microcystin Composition of the Cyanobacterium Planktothrix agardhii Changes toward a More Toxic Variant with Increasing Light Intensity. Appl. Environ. Microbiol..

[90] Briand E., Bormans M., Gugger M., Dorrestein P.C., Gerwick W.H. (2016). Changes in secondary metabolic profiles of Microcystis aeruginosa strains in response to intraspecific interactions. Environ. Microbiol..

[91] Hesse K., Dittmann E., Bornerm T. (2001). Consequences of impaired microcystin production for light-dependent growth and pigmentation of Microcystis aeruginosa PCC 7806. FEMS Microbiol. Ecol..

[92] Ferrão-Filho A.D.S., Kozlowsky-Suzuki B. (2011). Cyanotoxins: Bioaccumulation and Effects on Aquatic Animals. Mar. Drugs.

[93] Pearson L.A., Crosbie N.D., Neilan B.A. (2020). Distribution and conservation of known secondary metabolite biosynthesis gene clusters in the genomes of geographically diverse *Microcystis aeruginosa* strains. Mar. Freshw. Res..

[94] Teikari J., Österholm J., Kopf M., Battchikova N., Wahlsten M., Aro E.-M., Hess W.R., Sivonen K. (2015). Transcriptomic and Proteomic Profiling of Anabaena sp. Strain 90 under Inorganic Phosphorus Stress. Appl. Environ. Microbiol..

[95] Burberg C., Petzoldt T., von Elert E. (2020). Phosphate Limitation Increases Content of Protease Inhibitors in the Cyanobacterium *Microcystis aeruginosa*. Toxins.

[96] Pereira D.A., Pimenta A.M.C., Giani A. (2012). Profiles of toxic and non-toxic oligopeptides of *Radiocystis fernandoii* (Cyanobacteria) exposed to three different light intensities. Microbiol. Res..

[97] Seyedsayamdost M.R., Chandler J.R., Blodgett J.A.V., Lima P.S., Duerkop B.A., Oinuma K., Greenberg E.P., Clardy J. (2010). Quorum-sensing-regulated bactobolin production by Burkholderia thailandensis E264. Org. Lett..

[98] Guo X., Liu X., Wu L. (2016). The algicidal activity of *Aeromonas* sp. strain GLY-2107 against bloom-forming *Microcystis aeruginosa* is regulated by N-acyl homoserine lactone-mediated quorum sensing. Environ. Microbiol..

[99] Bähr L., Wüstenberg A., Ehwald R. (2016). Two-tier vessel for photoautotrophic high-density cultures. J. Appl. Phycol..

[100] Wells M.L., Potin P., Craigie J.S., Raven J.A., Merchant S.S., Helliwell K.E., Smith A.G., Camire M.E., Brawley S.H. (2017). Algae as nutritional and functional food sources: Revisiting our understanding. J. Appl. Phycol..

[101] Toporowska M., Mazur-Marzec H., Pawlik-Skowrońska B. (2020). The Effects of Cyanobacterial Bloom Extracts on the Biomass, Chl-a, MC and Other Oligopeptides Contents in a Natural Planktothrix agardhii Population. Int. J. Environ. Res. Public Health.

[102] Mutalipassi M., Riccio G., Mazzella V., Galasso C., Somma E., Chiarore A., de Pascale D., Zupo V. (2021). Symbioses of Cyanobacteria in Marine Environments: Ecological Insights and Biotechnological Perspectives. Mar. Drugs.

[103] Carpenter E.J., Foster R.A., Rai A.N., Bergman B., Rasmussen U. (2002). Marine cyanobacteria symbioses. Cyanobacteria in Symbiosis.

[104] Shridhar D.M.P., Mahajan G., Kamat V., Naik C., Parab R., Thakur N., Mishra P. (2009). Antibacterial Activity of 2-(2′,4′-Dibromophenoxy)-4,6-dibromophenol from *Dysidea granulosa*. Mar. Drugs.

[105] Finking R., Marahiel M.A. (2004). Biosynthesis of Nonribosomal Peptides. Annu. Rev. Microbiol..

[106] Süssmuth R.D., Mainz A. (2017). Nonribosomal Peptide Synthesis-Principles and Prospects. Angew. Chem. Int. Ed..

[107] Lima S.T., Alvarenga D.O., Etchegaray A., Fewer D.P., Jokela J., Varani A.M., Sanz M., Dörr F.A., Pinto E., Sivonen K. (2017). Genetic Organization of Anabaenopeptin and Spumigin Biosynthetic Gene Clusters in the Cyanobacterium *Sphaerospermopsis torques-reginae* ITEP-024. ACS Chem. Biol..

[108] Martínez-Núñez M.A., López V.E.L. (2016). Nonribosomal peptides synthetases and their applications in industry. Sustain. Chem. Process..

[109] Siezen R.J., Khayatt B.I. (2008). Natural products genomics. Microb. Biotechnol..

[110] Rouhiainen L., Jokela J., Fewer D.P., Urmann M., Sivonen K. (2010). Two Alternative Starter Modules for the Non-Ribosomal Biosynthesis of Specific Anabaenopeptin Variants in *Anabaena* (Cyanobacteria). Chem. Biol..

[111] Christiansen G., Philmus B., Hemscheidt T., Kurmayer R. (2011). Genetic Variation of Adenylation Domains of the Anabaenopeptin Synthesis Operon and Evolution of Substrate Promiscuity. J. Bacteriol..

[112] Kaljunen H., Schiefelbein S.H.H., Stummer D., Kozak S., Meijers R., Christiansen G., Rentmeister A. (2015). Structural Elucidation of the Bispecificity of A Domains as a Basis for Activating Non-natural Amino Acids. Angew. Chemie Int. Ed..

[113] Wilson M.C., Mori T., Rückert C., Uria A.R., Helf M.J., Takada K., Gernert C., Steffens U.A.E., Heycke N., Schmitt S. (2014). An environmental bacterial taxon with a large and distinct metabolic repertoire. Nature.

[114] Mareš J., Hájek J., Urajová P., Kust A., Jokela J., Saurav K., Galica T., Čapková K., Mattila A., Haapaniemi E. (2018). Alternative Biosynthetic Starter Units Enhance the Structural Diversity of Cyanobacterial Lipopeptides. Appl. Environ. Microbiol..

[115] Imker H.J., Walsh C.T., Wuest W.M. (2009). SylC Catalyzes Ureido-Bond Formation During Biosynthesis of the Proteasome Inhibitor Syringolin A. J. Am. Chem. Soc..

[116] Koketsu K., Mitsuhashi S., Tabata K. (2013). Identification of Homophenylalanine Biosynthetic Genes from the Cyanobacterium Nostoc punctiforme PCC73102 and Application to Its Microbial Production by *Escherichia coli*. Appl. Environ. Microbiol..

[117] Khumalo M.J., Nzuza N., Padayachee T., Chen W., Yu J.-H., Nelson D.R., Syed K. (2020). Comprehensive Analyses of Cytochrome P450 Monooxygenases and Secondary Metabolite Biosynthetic Gene Clusters in *Cyanobacteria*. Int. J. Mol. Sci..

[118] Mazur-Marzec H., Sutryk K., Hebel A., Hohlfeld N., Pietrasik A., Błaszczyk A. (2015). Nodularia spumigena Peptides—Accumulation and Effect on Aquatic Invertebrates. Toxins.

[119] Agha R., Gross A., Rohrlack T., Wolinska J. (2018). Adaptation of a chytrid parasite to its cyanobacterial host is hampered by host intraspecific diversity. Front. Microbiol..

[120] Ger K.A., Urrutia-Cordero P., Frost P.C., Hansson L.A., Sarnelle O., Wilson A.E., Lürling M. (2016). The interaction between cyanobacteria and zooplankton in a more eutrophic world. Harmful Algae.

[121] Kyle M., Haande S., Ostermaier V., Rohrlack T. (2015). The Red Queen Race between Parasitic Chytrids and Their Host, Planktothrix: A Test Using a Time Series Reconstructed from Sediment DNA. PLoS ONE.

[122] Urrutia-Cordero P., Agha R., Cirés S., Lezcano M.Á., Sánchez-Contreras M., Waara K.-O., Utkilen H., Quesada A. (2013). Effects of harmful cyanobacteria on the freshwater pathogenic free-living amoeba *Acanthamoeba castellanii*. Aquat. Toxicol..

[123] Rohrlack T., Christoffersen K., Kaebernick M., Neilan B.A. (2004). Cyanobacterial Protease Inhibitor Microviridin J Causes a Lethal Molting Disruption in *Daphnia pulicaria*. Appl. Environ. Microbiol..

[124] Schwarzenberger A., Sadler T., Von Elert E. (2013). Effect of nutrient limitation of cyanobacteria on protease inhibitor production and fitness of *Daphnia magna*. J. Exp. Biol..

[125] Papadimitriou T., Kagalou I., Stalikas C., Pilidis G., Leonardos I.D. (2012). Assessment of microcystin distribution and biomagnification in tissues of aquatic food web compartments from a shallow lake and evaluation of potential risks to public health. Ecotoxicology.

[126] Leonard J.A., Paerl H.W. (2005). Zooplankton community structure, micro-zooplankton grazing impact, and seston energy content in the St. Johns river system, Florida as influenced by the toxic cyanobacterium *Cylindrospermopsis raciborskii*. Hydrobiologia.

[127] Pawlik-Skowrońska B., Toporowska M., Mazur-Marzec H. (2019). Effects of secondary metabolites produced by different cyanobacterial populations on the freshwater zooplankters *Brachionus calyciflorus* and *Daphnia pulex*. Environ. Sci. Pollut. Res..

[128] Moustaka-Gouni M., Sommer U. (2020). Effects of Harmful Blooms of Large-Sized and Colonial Cyanobacteria on Aquatic Food Webs. Water.

[129] Holland A., Kinnear S. (2013). Interpreting the Possible Ecological Role(s) of Cyanotoxins: Compounds for Competitive Advantage and/or Physiological Aide?. Mar. Drugs.

[130] Roegner A., Truong L., Weirich C., Schirmer M.P., Brena B., Miller T.R., Tanguay R. (2019). Combined Danio rerio embryo morbidity, mortality and photomotor response assay: A tool for developmental risk assessment from chronic cyanoHAB exposure. Sci. Total Environ..

[131] Lenz K.A., Miller T.R., Ma H. (2019). Anabaenopeptins and cyanopeptolins induce systemic toxicity effects in a model organism the nematode *Caenorhabditis elegans*. Chemosphere.

[132] Muszewska A., Stepniewska-Dziubinska M.M., Steczkiewicz K., Pawlowska J., Dziedzic A., Ginalski K. (2017). Fungal lifestyle reflected in serine protease repertoire. Sci. Rep..

[133] Dehm D., Krumbholz J., Baunach M., Wiebach V., Hinrichs K., Guljamow A., Tabuchi T., Jenke-Kodama H., Süssmuth R.D., Dittmann E. (2019). Unlocking the Spatial Control of Secondary Metabolism Uncovers Hidden Natural Product Diversity in Nostoc punctiforme. ACS Chem. Biol..

[134] Mazur-Marzec H., Toruńska A., Błońska M.J., Moskot M., Pliński M., Jakóbkiewicz-Banecka J., Węgrzyn G. (2009). Biodegradation of nodularin and effects of the toxin on bacterial isolates from the Gulf of Gdańsk. Water Res..

[135] Kansole M., Lin T.-F. (2016). Microcystin-LR Biodegradation by Bacillus sp.: Reaction Rates and Possible Genes Involved in the Degradation. Water.

[136] Seymour J.R., Amin S.A., Raina J.-B., Stocker R. (2017). Zooming in on the phycosphere: The ecological interface for phytoplankton–bacteria relationships. Nat. Microbiol..

[137] Briand E., Humbert J., Tambosco K., Bormans M., Gerwick W.H. (2016). Role of bacteria in the production and degradation of *Microcystis cyanopeptides*. Microbiologyopen.

[138] Kato H., Imanishi S.Y., Tsuji K., Harada K. (2007). Microbial degradation of cyanobacterial cyclic peptides. Water Res..

[139] Kurmayer R., Deng L., Entfellner E. (2016). Role of toxic and bioactive secondary metabolites in colonization and bloom formation by filamentous cyanobacteria *Planktothrix*. Harmful Algae.

[140] Šulčius S., Mazur-Marzec H., Vitonytė I., Kvederavičiūtė K., Kuznecova J., Šimoliūnas E., Holmfeldt K. (2018). Insights into cyanophage-mediated dynamics of nodularin and other non-ribosomal peptides in *Nodularia spumigena*. Harmful Algae.

[141] Mazur-Marzec H., Błaszczyk A., Felczykowska A., Hohlfeld N., Kobos J., Toruńska-Sitarz A., Devi P., Montalvão S., D’souza L., Tammela P. (2015). Baltic cyanobacteria—A source of biologically active compounds. Eur. J. Phycol..

[142] Ahmad S., Saleem M., Riaz N., Lee Y.S., Diri R., Noor A., Almasri D., Bagalagel A., Elsebai M.F. (2020). The Natural Polypeptides as Significant Elastase Inhibitors. Front. Pharmacol..

[143] Ammosova T., Jerebtsova M., Beullens M., Voloshin Y., Ray P.E., Kumar A., Bollen M., Nekhai S. (2003). Nuclear Protein Phosphatase-1 Regulates HIV-1 Transcription. J. Biol. Chem..

[144] McConnell J.L., Wadzinski B.E. (2009). Targeting Protein Serine/Threonine Phosphatases for Drug Development. Mol. Pharmacol..

[145] Halland N., Brönstrup M., Czech J., Czechtizky W., Evers A., Follmann M., Kohlmann M., Schiell M., Kurz M., Schreuder H.A. (2015). Novel Small Molecule Inhibitors of Activated Thrombin Activatable Fibrinolysis Inhibitor (TAFIa) from Natural Product Anabaenopeptin. J. Med. Chem..

[146] Hameed S. (2013). Investigation of the Production and Isolation from Cyanobacteria.

[147] Gulledge B., Aggen J., Huang H., Nairn A., Chamberlin A. (2002). The Microcystins and Nodularins: Cyclic Polypeptide Inhibitors of PP1 and PP2A. Curr. Med. Chem..

[148] Bubik A., Sedmak B., Novinec M., Lenarčič B., Lah T.T. (2008). Cytotoxic and peptidase inhibitory activities of selected non-hepatotoxic cyclic peptides from cyanobacteria. Biol. Chem..

[149] Vercauteren E., Gils A., Declerck P. (2013). Thrombin Activatable Fibrinolysis Inhibitor: A Putative Target to Enhance Fibrinolysis. Semin. Thromb. Hemost..

[150] Shah S., Akhter N., Auckloo B., Khan I., Lu Y., Wang K., Wu B., Guo Y.-W. (2017). Structural Diversity, Biological Properties and Applications of Natural Products from Cyanobacteria. A Review. Mar. Drugs.

[151] Wahome P., Beauchesne K., Pedone A., Cavanagh J., Melander C., Zimba P., Moeller P. (2014). Augmenting Anti-Cancer Natural Products with a Small Molecule Adjuvant. Mar. Drugs.

[152] Shishido T.K., Popin R.V., Jokela J., Wahlsten M., Fiore M.F., Fewer D.P., Herfindal L., Sivonen K. (2019). Dereplication of Natural Products with Antimicrobial and Anticancer Activity from Brazilian Cyanobacteria. Toxins.

