# Supplementary material for: Anabaenopeptins: What We Know So Far
Source: Toxins (Basel). 2021 Jul 27;13(8):522. doi: 10.3390/toxins13080522 (PMC8402340; doi:10.3390/toxins13080522)
Supplement: Supplementary file 1 [file toxins-13-00522-s001.zip › toxins-1182298-supplementary.pdf]

Anabaenopeptins: What We Know So Far

Patrick Romano Monteiro, Samuel Cavalcante do Amaral, Andrei Santos Siqueira, Luciana Pereira Xavier and Agenor Valadares Santos

Table S1. Sequence composition of Anabaenopeptins. Amino acids are considered in L-configuration, unless otherwise defined.

Nomenclature		Position						References
		1	2	3	4	5	6	
Anabaenopeptin	679	-	D-Lys	Val	Hty	MeAla	Phe	[53]
Anabaenopeptin	731	Phe	D-Lys	Val	Leu	MeGly	AcSer	[81]
Anabaenopeptin	788	Val	D-Lys	Val	Trp	MeAla	Phe	[74]
Anabaenopeptin	803	Leu	D-Lys	Met	Leu	MeIle	MetO	[81]
Anabaenopeptin	807	Ile	D-Lys	Ile	Hty	MeAla	Phe	[14,34]
Anabaenopeptin	813	Phe	D-Lys	Val	Hty	MeGly	Phe	[34]
Anabaenopeptin	816	Leu	D-Lys	Leu	Trp	MeAla	Phe	[74]
Anabaenopeptin	828	Tyr	D-Lys	Val	Hph	MeAla	Phe	[75]
Anabaenopeptin	842	Tyr	D-Lys	Ile	Hph	MeAla	Phe	[75]
Anabaenopeptin	848	Leu	D-Lys	Ile	MeHph	MeAsn	Phe	[24]
Anabaenopeptin	849	Lys	D-Lys	Ile	Hph	MeAsn	Phe	[24]
Anabaenopeptin	852	MeHty	D-Lys	Val	Hty	MeAla	MeLeu	[84]
Anabaenopeptin	855	Phe	D-Lys	Ile	Hph	MeAla	Hty	[24]
Anabaenopeptin	856	MeHty	D-Lys	Val	Hph	MeAla	Phe	[84]
Anabaenopeptin	857	Phe	D-Lys	Val	Hty	MeAla	Hty	[24]
Anabaenopeptin	862	Leu	D-Lys	Ile	EtHph	MeAsn	Phe	[24]
Anabaenopeptin	863	Lys	D-Lys	Ile	MeHph	MeAsn	Phe	[24]
Anabaenopeptin	866	MeHty	D-Lys	Ile	Hty	MeAla	MeLeu	[84]
Anabaenopeptin	870	MeHty	D-Lys	Ile	Hph	MeAla	Phe	[84]
Anabaenopeptin	871	Phe	D-Lys	Ile	Hty	MeAla	Hty	[24]
Anabaenopeptin	872	MeHty	D-Lys	Val	Hty	MeAla	Phe	[84]
Anabaenopeptin	882	Phe	D-Lys	Ile	MeHph	MeAsn	Phe	[24]
Anabaenopeptin	886	MeHty	D-Lys	Ile	Hty	MeAla	Phe	[84]
Anabaenopeptin	891	Arg	D-Lys	Ile	MeHph	MeAsn	Phe	[24]
Anabaenopeptin	896	Phe	D-Lys	Ile	EtHph	MeAsn	Phe	[24]
Anabaenopeptin	899	Phe	D-Lys	Val	Hty	MeHty	Ile	[70]
Anabaenopeptin	900	MeHty	D-Lys	Ile	Hty	MeAla	MePhe	[84]
Anabaenopeptin	905	Arg	D-Lys	Ile	EtHph	MeAsn	Phe	[24]
Anabaenopeptin	915	Tyr	D-Lys	Val	Hty	MeHty	Ile	[62,63,76]
Anabaenopeptin	802A	Val	D-Lys	Ile	Trp	MeAla	Phe	[24,74]
Anabaenopeptin	802B	Val	D-Lys	Leu	Trp	MeAla	Phe	[74]
Anabaenopeptin	841A	Phe	D-Lys	Ile	Hty	MeAla	Phe	[14]
Anabaenopeptin	841B	Phe	D-Lys	Val	Hph	MeAla	Hty	[24]
Anabaenopeptin	877B	Lys	D-Lys	Ile	EtHph	MeAsn	Phe	[24]
Anabaenopeptin	877A	Arg	D-Lys	Ile	Hph	MeAsn	Phe	[24]
Anabaenopeptin	908A	Arg	D-Lys	Val	Hty	MeHty	Ile	[62,63,83]
Anabaenopeptin	908B	Arg	D-Lys	Val	Hty	MeHty	Leu	[53]
Anabaenopeptin	A	Tyr	D-Lys	Val	Hty	MeAla	Phe	[44]
Anabaenopeptin	B	Arg	D-Lys	Val	Hty	MeAla	Phe	[44,48]
Anabaenopeptin	C	Lys	D-Lys	Val	Hty	MeAla	Phe	[44]
Anabaenopeptin	D	Phe	D-Lys	Val	Hty	MeAla	Phe	[44]

Anabaenopeptin	E	Arg	D-Lys	Val	MeHty	MeAla	Phe	[38]
Anabaenopeptin	F	Arg	D-Lys	Ile	Hty	MeAla	Phe	[38]
Anabaenopeptin	G	Tyr	D-Lys	Ile	Hty	MeHty	Ile	[26]
Anabaenopeptin	H	Arg	D-Lys	Ile	Hty	MeHty	Ile	[26]
Anabaenopeptin	HU892	Arg	D-Lys	Val	Hph	MeHty	Ile	[61]
Anabaenopeptin	I	Ile	D-Lys	Val	Hty	MeAla	Leu	[42]
Anabaenopeptin	J	Ile	D-Lys	Val	Hty	MeAla	Phe	[42]
Anabaenopeptin	KB899	Tyr	D-Lys	Val	Hph	MeHty	Ile	[62]
Anabaenopeptin	KB906	Arg	D-Lys	Ile	Hph	MeHty	Ile	[62]
Anabaenopeptin	KT864	Harg	D-Lys	Ile	Hty	MeAla	Phe	[52]
Anabaenopeptin	KVJ811	Phe	D-Lys	Val	Hph	MeGly	Hph	[21]
Anabaenopeptin	KVJ827	Tyr	D-Lys	Val	Hph	MeGly	Hph	[21]
Anabaenopeptin	KVJ841	Tyr	D-Lys	Ile	Hph	MeGly	Hph	[21]
Anabaenopeptin	MM823	O-MeGlu	D-Lys	Val	Hty	MeAla	Phe	[22]
Anabaenopeptin	MM850	O-MeArg	D-Lys	Val	Hty	MeAla	Phe	[22]
Anabaenopeptin	MM913	Tyr	D-Lys	Ile	Hph	MeHty	Ile	[22]
Anabaenopeptin	NZ825	Phe	D-Lys	Ile	Hph	MeGly	Hph	[71]
Anabaenopeptin	NZ841	Phe	D-Lys	Ile	Hph	MeGly	Hty	[71,83]
Anabaenopeptin	NZ857	Phe	D-Lys	Ile	Hty	MeGly	Hty	[71]
Anabaenopeptin	SA1	Arg	D-Lys	Ile	PNV	Asn	Phe	[12]
Anabaenopeptin	SA10	Phe	D-Lys	Val	Hty	Gly	Cl-Hty	[12]
Anabaenopeptin	SA11	Phe	D-Lys	Ile	Hty	Gly	Cl-Hty	[12]
Anabaenopeptin	SA12	Phe	D-Lys	Val	Hty	Gly	Hty	[12]
Anabaenopeptin	SA13	Tyr	D-Lys	Val	Hty	Ser	Phe	[12]
Anabaenopeptin	SA2	Arg	D-Lys	Val	Hty	Ser	Phe	[12]
Anabaenopeptin	SA3	Lys	D-Lys	Ile	Hty	Ala	Phe	[12]
Anabaenopeptin	SA4	Lys	D-Lys	Ile	PNV	Asn	Phe	[12]
Anabaenopeptin	SA5	Ile	D-Lys	Val	PNV	Asn	Phe	[12]
Anabaenopeptin	SA6	Ile	D-Lys	Ile	Hph	Asn	Phe	[12]
Anabaenopeptin	SA7	Ile	D-Lys	Ile	PNV	Asn	Phe	[12]
Anabaenopeptin	SA8	Ile	D-Lys	Ile	PNL	Asn	Phe	[12]
Anabaenopeptin	SA9	Phe	D-Lys	Ile	Cl-Hty	Gly	Hph	[12]
Anabaenopeptin	T	Ile	D-Lys	Val	Hty	MeHty	Ile	[41]
Brunsvicamides	A	Ile	Lys	Val	Leu	MeTrp	Phe	[50]
Brunsvicamides	B	Ile	Lys	Ile	Leu	MeTrp	Phe	[50]
Brunsvicamides	C	Ile	Lys	Val	Leu	MeFormyl-kynurenine	Phe	[50]
Ferintoic Acid	A	Trp	D-Lys	Val	Hty	MeAla	Phe	[39]
Ferintoic Acid	B	Trp	D-Lys	Allo-Ile	Hty	MeAla	Phe	[39]
Ferintoic Acid	C	Trp	D-Lys	Met	Hty	MeAla	Phe	[78]
Ferintoic Acid	D	Trp	D-Lys	MetO	Hty	MeAla	Phe	[78]
Lyngbyaureidamide	A	D-Phe	D-Lys	Ile	Hty	MeAla	Hph	[47]
Lyngbyaureidamide	B	D-Phe	D-Lys	Ile	Hty	MeAla	Phe	[47]
Namalide	B	Ile	D-Lys	Ile	Hty	-	-	[73]
Namalide	C	Ile	D-Lys	Val	Hty	-	-	[73]
Nodulapeptin	807	Ile	D-Lys	Val	Hph	MeHph	Ser	[49]
Nodulapeptin	821	Ile	D-Lys	Ile	Hph	MeHph	Ser	[49]
Nodulapeptin	823	Ile	D-Lys	Val	Hph	MeHty	Ser	[49]
Nodulapeptin	839	Ile	D-Lys	Met	Hph	MeHph	Ser	[49]
Nodulapeptin	849	Ile	D-Lys	Val	Hph	MeHph	AcSer	[49]
Nodulapeptin	851	Ile	D-Lys	Val	Hph	MeHph	Met	[49]

Nodulapeptin	857	Phe	D-Lys	Val	Hph	MeHty	Ser	[34,49]
Nodulapeptin	863	Ile	D-Lys	Ile	Hph	MeHph	AcSer	[49]
Nodulapeptin	865	Ile	D-Lys	Val	Hph	MeHty	AcSer	[34]
Nodulapeptin	867	Ile	D-Lys	Val	Hph	MeHty	Met	[34]
Nodulapeptin	869	Phe	D-Lys	Val	Leu	MeHty	MetO	[34]
Nodulapeptin	871	Ile	D-Lys	MetO	Hph	MeHty	Ser	[14,73]
Nodulapeptin	879	Ile	D-Lys	Ile	Hph	MeHty	AcSer	[14]
Nodulapeptin	885	Phe	D-Lys	Val	Hph	MeHph	Met	[49]
Nodulapeptin	897	Ile	D-Lys	MetO	Hph	MeHph	AcSer	[14,73]
Nodulapeptin	901	Phe	D-Lys	Val	Hph	MeHty	Met	[14]
Nodulapeptin	917	Phe	D-Lys	Val	Hph	MeHty	MetO	[14,34]
Nodulapeptin	931	Ile	D-Lys	MetO	Hph	MeHty	MetO	[14,34]
Nodulapeptin	933	Phe	D-Lys	Val	Hty	MeHty	MetO	[14,34]
Nodulapeptin	855A	Ile	D-Lys	Met	Hph	MeHty	Ser	[14]
Nodulapeptin	855B	Ile	D-Lys	MetO	Hph	MeHph	Ser	[14]
Nodulapeptin	855C	Phe	D-Lys	Val	Val	MeHty	MetO	[34]
Nodulapeptin	881A	Ile	D-Lys	Met	Hph	MeHph	AcSer	[14,34]
Nodulapeptin	881B	Ile	D-Lys	Ile	Hph	MeHty	Met	[14,34]
Nodulapeptin	883A	Ile	D-Lys	Met	Hph	MeHph	Met	[14,85]
Nodulapeptin	883B	Phe	D-Lys	Val	Hph	MeHph	AcSer	[14]
Nodulapeptin	883C	Ile	D-Lys	Val	Hph	MeHty	MetO	[34]
Nodulapeptin	899A	Ile	D-Lys	Met	Hph	MeHty	Met	[14,34]
Nodulapeptin	899B	Phe	D-Lys	Val	Hph	MeHty	AcSer	[14,34]
Nodulapeptin	899C	Ile	D-Lys	Val	Hty	MeHty	MetO	[34]
Nodulapeptin	915A	Ile	D-Lys	MetO	Hph	MeHty	Met	[14,73]
Nodulapeptin	915B	Phe	D-Lys	Val	Hty	MeHty	AcSer	[14]
Nodulapeptin	A	Ile	D-Lys	MetO2	Hph	MeHty	AcSer	[48]
Nodulapeptin	B	Ile	D-Lys	MetO	Hph	MeHty	AcSer	[14,48]
Nodulapeptin	C	Ile	D-Lys	Met	Hph	MeHty	AcSer	[14]
Oscillamide	B	Arg	D-Lys	Met	Hty	MeAla	Phe	[25]
Oscillamide	Y	Tyr	D-Lys	Ile	Hty	MeAla	Phe	[36]
Pompanopeptin	B	Ile	Lys	Val	Hty	MeAhpha	Hty	[23]
Schizopeptin	791	Ile	D-Lys	Ile	Hph	MeAla	Phe	[46]

AcSer: Acetyl Serine; Ala: Alanine; Allo-Ile: Allo-isoleucine; Arg: Arginine; Asn: Asparagine; Cl-Hty: 2-Chloro-Homotyrosine; D-Phe: D-Phenylalanine; EtHph: N-Ethyl-Homophenylalanine; Gly: Glycine; Harg: Homoarginine; Hph: Homophenylalanine; Hty: Homotyrosine; Ile: Isoleucine; Leu: Leucine; Lys: Lysine; MeAhpha: N-Methyl-2-amino-6-(40-hydroxyphenyl) hexanoic acid; MeAla: Methyl-Alanine; MeAsn: Methyl-Asparagine; MeFormyl-kynurenine: N-Methyl-N′-formyl-D-kynurenine; MeGly: Methyl Glycine; MeHph: Methyl-Homophenylalanine; MeHty: Methyl-Tyrosine; MeIle: Methyl-Isoleucine; MeLeu: Methyl-Leucine; MePhe: Methyl-Phenylalanine; Met: Methionine; MetO: Methionine sulfoxide; MetO2: Methionine sulfone; MeTrp: Methyl-Tryptophan; O-MeArg: O-Methyl-Arginine; O-MeGlu: Methoxy-Glutamate; Phe: Phenylalanine; PNL: 6-Phenylnorleucine; PNV: 5-Phenylnorvaline; Ser: Serine; Trp: Tryptophan; Tyr: Tyrosine and Val: Valine.
